# The Glycemic Curve during the Oral Glucose Tolerance Test: Is It Only Indicative of Glycoregulation?

**DOI:** 10.3390/biomedicines11051278

**Published:** 2023-04-25

**Authors:** Daniela Vejrazkova, Marketa Vankova, Petra Lukasova, Martin Hill, Josef Vcelak, Andrea Tura, Denisa Chocholova, Bela Bendlova

**Affiliations:** 1 Institute of Endocrinology, 110 00 Prague, Czech Republicplukasova@endo.cz (P.L.); mhill@endo.cz (M.H.); bbendlova@endo.cz (B.B.); 2 Institute of Neuroscience, National Research Council (CNR), 351 22 Padova, Italy; andrea.tura@cnr.it; 3 Faculty of Science, Charles University, 128 00 Prague, Czech Republic; denisa.chocholova@natur.cuni.cz

**Keywords:** glycemic curve, glucose curve shape, delayed glucose peak, insulin sensitivity, beta cell function, oral glucose tolerance test, glucose tolerance, type 2 diabetes mellitus

## Abstract

The shape of the glycemic curve during the oral glucose tolerance test (OGTT), interpreted in the correct context, can predict impaired glucose homeostasis. Our aim was to reveal information inherent in the 3 h glycemic trajectory that is of physiological relevance concerning the disruption of glycoregulation and complications beyond, such as components of metabolic syndrome (MS). Methods: In 1262 subjects (1035 women, 227 men) with a wide range of glucose tolerance, glycemic curves were categorized into four groups: monophasic, biphasic, triphasic, and multiphasic. The groups were then monitored in terms of anthropometry, biochemistry, and timing of the glycemic peak. Results: Most curves were monophasic (50%), then triphasic (28%), biphasic (17.5%), and multiphasic (4.5%). Men had more biphasic curves than women (33 vs. 14%, respectively), while women had more triphasic curves than men (30 vs. 19%, respectively) (*p* < 0.01). Monophasic curves were more frequent in people with impaired glucose regulation and MS compared to bi-, tri-, and multiphasic ones. Peak delay was the most common in monophasic curves, in which it was also most strongly associated with the deterioration of glucose tolerance and other components of MS. Conclusion: The shape of the glycemic curve is gender dependent. A monophasic curve is associated with an unfavorable metabolic profile, especially when combined with a delayed peak.

## 1. Introduction

### 1.1. Pathogenesis of Impaired Glucose Homeostasis and Diabetes Mellitus

Diabetes mellitus belongs to one of the most widespread civilization diseases. The largest group of diabetic people is represented by patients with type 2 diabetes mellitus (T2DM). T2DM is a chronic metabolic disorder that is characterized by hyperglycemia in the context of insulin resistance (IR) and relative lack of insulin. Long-lasting periods of impaired fasting glucose (IFG) and/or impaired glucose tolerance (IGT) usually precedes T2DM manifestations. T2DM is associated with a ten-year-shorter life expectancy, especially due to serious micro- and macrovascular complications, including coronary artery disease, strokes, diabetic retinopathy, kidney failure, and poor blood flow in limbs leading to amputations. In the Czech Republic, the prevalence of T2DM reaches 8–9%, with an alarming rise in younger age groups, probably mainly due to lifestyle changes and the increasing occurrence of obesity [1,2].

The pathogenesis of IFG, IGT, IR, and T2DM is complex. IR, as well as decreased beta cell function, can be a consequence of impaired glucose and lipid metabolism, impaired energy homeostasis, the excess and/or impaired function of adipose tissue, gastrointestinal hormonal dysfunction, an altered gut microbiome, chronic inflammation and, of course, a consequence of all those components combined [3,4]. Although external factors, such as diet, physical activity, stress, polluted environments, endocrine disruptors, or even conditions of prenatal development and birth weight [5,6,7,8,9], play important roles in the pathogenesis of IGT, IR, and T2DM, there is evidence that the disease is strongly genetically determined [10,11]. The development of the overt disease then reflects an interaction with a wide variety of developmental and external factors with genetic backgrounds [12]. Thus, diabetes is a typically complex, polygenic, and multifactorial disease [13,14]. Genome-wide association studies (GWAS) and their meta-analyses established 65 independent European-derived loci associated with T2DM and 36 loci contributing to variations in fasting plasma glucose. However, the effect size of the individual genetic loci on either T2DM risk or fasting plasma glucose was modest, and these SNPs explained less than 10% of T2DM heritability and less than 5% of fasting plasma glucose variance [15,16]. Thus, the clinical utility of these loci for predicting T2DM is still a matter of debate [17].

The oral glucose tolerance test (OGTT) reflects the dynamics of response to glucose stimulus and is widely used in clinical practice as a diagnostic test to detect impaired glucose tolerance and T2DM. However, interpretation limited to diagnostic criteria defined at the 0 and 120th min of the test may miss some physiologically important information. When interpreted correctly in a relevant context, this test could have much wider clinical utility, including the capability to predict the development of glucose intolerance and other related disorders.

### 1.2. Individual Response to OGTT

There are currently several approaches for assessing beta cell function and insulin sensitivity (IS) in vivo [18]. Among them, the OGTT and the derived equations (indices of IS, and insulin secretion and disposition indices based on the measurements of glucose, insulin, and C-peptide during the OGTT) are being used in various clinical settings as the most suitable methods for epidemiological studies [19].

As already mentioned, the course of the glycemic curve can predict the possible future development of IGT or later progression to overt T2DM. Indeed, many scientific teams have been dealing with this topic in recent years [20,21,22]. Studies evaluating the shape of the curve during the 2 h OGTT predominate, but studies evaluating the 3 h OGTT shape are no longer rare. In more detail, the shape is defined by the pattern of rising and falling glucose concentrations after a standard 75 g glucose load as monophasic, biphasic, triphasic, or multiphasic [23]. The monophasic curve has been associated in the literature with lower IS and decreased beta cell function [20,21]. Higher insulin resistance and impaired beta cell secretion have also been associated with a delay in the time of the first peak of glycemia and a rising glucose level at the peak [22]. Therefore, we will pay special attention to the time when blood glucose culminates.

### 1.3. Study Aims

Our aim was to evaluate and describe the variability in the shape of the 3 h OGTT glycemic curves in a large cohort of people, including healthy individuals, people with impaired fasting glucose and impaired glucose tolerance, and newly diagnosed type 2 diabetics. We aimed to characterize specific groups defined by the shape of the glycemic curve in order to reveal the information inherent in the glycemic trajectory that is of physiological or even clinical relevance for the early detection and subsequent prevention of metabolic disturbances and complications. Such stratification would be especially beneficial for individuals with fasting and post-challenge blood glucose at the 120th min of the OGTT within the normal range, who may escape medical attention but, nevertheless, are more likely to develop metabolic disorders, of which they should be aware. Such people can be thoroughly educated in terms of lifestyle and benefit from possible early intervention.

## 2. Materials and Methods

### 2.1. Study Subjects

In the years 2001–2022, adult Czech individuals with varying degrees of glucose tolerance were continuously examined at the Institute of Endocrinology in Prague. Examinations were based on anthropometric and biochemical characterization, including the 3 h OGTT. The cohort consisted of completely healthy individuals, individuals with glucose metabolism disorders, and newly diagnosed type 2 diabetics. Exclusion criteria included serious diseases, where undergoing a glucose test would pose a health risk, pregnancy, or ages below 18 and above 75 years. Complete biochemical and anthropometric characterization was performed in 1262 individuals, comprising 1035 women (age of 34.7 ± 10.25 years, mean ± standard deviation) and 227 men (age of 36.5 ± 13.13 years, mean ± standard deviation). Participants were asked about their medications. Among the medications relevant to the parameters assessed, 53 (4.2%) reported treatment of hypertension and 46 (3.6%) of dyslipidemia. Such low percentages of treated people can be explained mainly by the relatively low average age. None of the participants had been treated for T2DM; diabetic patients in our cohort were newly diagnosed based on our examination. The study protocol was in accordance with the institutional ethics committee (Ethics Committee of the Institute of Endocrinology EK_EÚ_10062019). All participants signed an informed consent, in which they were properly informed about the course of the examination and had the opportunity to ask questions related to their participation in the study.

### 2.2. Metabolic and Anthropometric Characterization of the Subjects

Venous blood samples were taken at 8 a.m. after overnight fasting. Glucose metabolism was characterized by blood glucose (enzymatic reference method with hexokinase, Roche, Cobas 6000, Roche Diagnostic, Mannheim), insulin (ECLIA, Roche, Cobas 6000, Roche Diagnostic, Mannheim), and C-peptide (ECLIA, Roche, Cobas 6000, Roche Diagnostic, Mannheim). During the 3 h OGTT (75 g of glucose in 250 mL of water), trajectories of blood glucose, insulin, and C-peptide were analyzed according to Tura et al. [23] with sampling every 30 min of the test (i.e., at 0, 30, 60, 90, 120, 150, and 180 min) using cannula. Areas under the glycemic (AUC gluc), insulin (AUC ins), and C-peptide curves (AUC cp) were calculated based on the trapezoidal rule. To assess insulin sensitivity (IS), indices, such as homeostasis model HOMA-R or its logarithmically transformed version QUICKI, were calculated for the fasting condition, as well as ISIcomp (also known as Matsuda’s index), OGIS (both 2 h and 3 h), MCRest, Si (oral), and PREDIM in the dynamic conditions following the glucose load [24,25]. Beta cell function was evaluated using HOMA-beta during fasting state and indices based both on insulin and C-peptide, such as the insulinogenic index IGI and its C-peptide-based version in dynamics, IGI cp [26]. In addition, we computed the oral disposition indices (DIs). DI represents an index of beta cell function related to IS and was calculated as IGI × ISIcomp as well as according to the OGIS 3h × AUC ins formula [27]. In parallel, using C-peptide, adaptation index (AI) was calculated as OGIS 3h × AUC cp [28]. For dynamic indices, we also assessed the early phase of insulin and C-peptide secretion (calculated from the first 30 min of the OGTT). Details of the tabulated indices, including units, are given in Appendix A, Table A1.

Lipid profile was evaluated by total cholesterol (enzymatic colorimetric test, Roche, Cobas 6000, Roche Diagnostic, Mannheim), high-density lipoprotein (HDL) cholesterol (homogeneous enzymatic colorimetric test, Roche, Cobas 6000, Roche Diagnostic, Mannheim), low-density lipoprotein (LDL) cholesterol (homogeneous enzymatic colorimetric test, Roche, Cobas 6000, Roche Diagnostic, Mannheim), and triacylglycerol concentrations (enzymatic colorimetric test, Roche, Cobas 6000, Roche Diagnostic, Mannheim).

Furthermore, thyroid hormones TSH, free T3, free T4 (ECLIA; Cobas 6000, Roche Diagnostics, Mannheim, Germany) and liver enzymes ALT, AST, and GGT (IFCC method with pyridoxal phosphate; Cobas 6000, Roche Diagnostics, Mannheim, Germany) were assessed. Hepatic insulin extraction (HE) was evaluated according to Tura et al. [23].

Systolic and diastolic blood pressures were measured in resting state.

Body height and weight were determined to calculate body mass index (BMI), whereas waist and hip circumferences were measured in order to calculate waist-to-hip ratio (WHR) and evaluate body fat distribution. Furthermore, body adiposity index (BAI) was determined to estimate the amount of body fat [29]. Metabolic syndrome was diagnosed according to NCEP_ATPIII criteria [30] and PCOS according to the European Society of Human Reproduction and Embryology consensus [31].

### 2.3. Classification of the OGTT Curves

The shape of the glucose curve was classified as monophasic if glycemia simply increased and then gradually decreased (one peak). The shape was biphasic if the blood glucose showed a further increase after a previous decrease. The triphasic shape was characterized by two complete peaks. During the 3 h OGTT, much more complex and heterogeneous curve shapes were observed. In some people, there were also four- and five-phase curves with three and four complete peaks, respectively. These were pooled as multiphasic type of glycemic curves.

**Figure 1:**
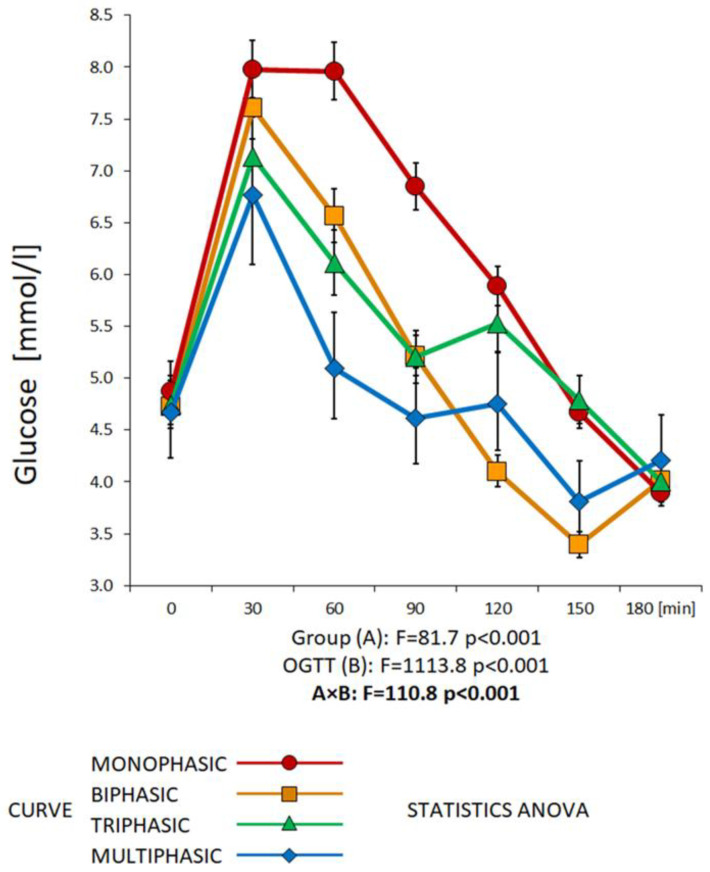
Illustration of the types of glycemic curve shapes during the 3 h oral glucose tolerance test (OGTT).

Glucose variations were considered significant if the difference was at least 2% (this criterion was necessary to avoid the detection of false minima and maxima in the glucose curve). Higher requirement for significant variability (5%) was chosen for the insulin and C-peptide curves [23]. Figure 1 illustrates the morphology of the different types of glycemic trajectories.

### 2.4. Statistical Evaluation

**Figure 2:**
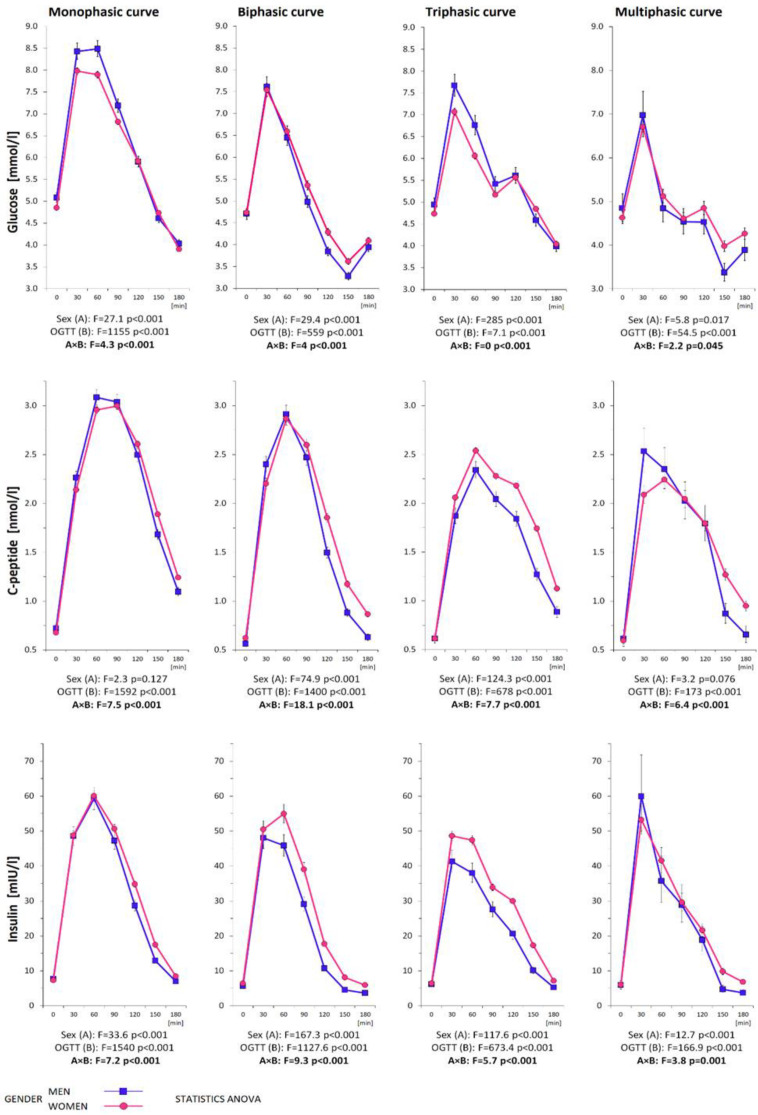
The course of glycemic, insulinemic and C-peptide curves during the 3 h OGTT for women and men.

**Figure 3:**
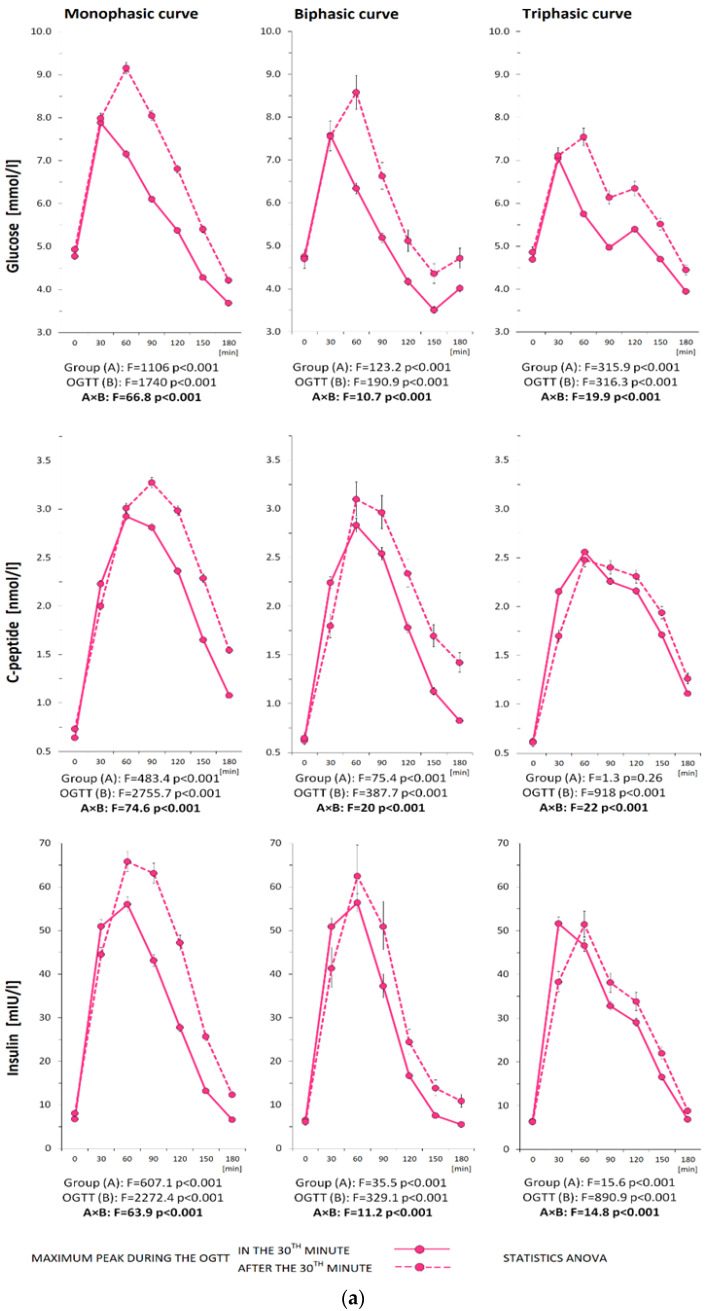

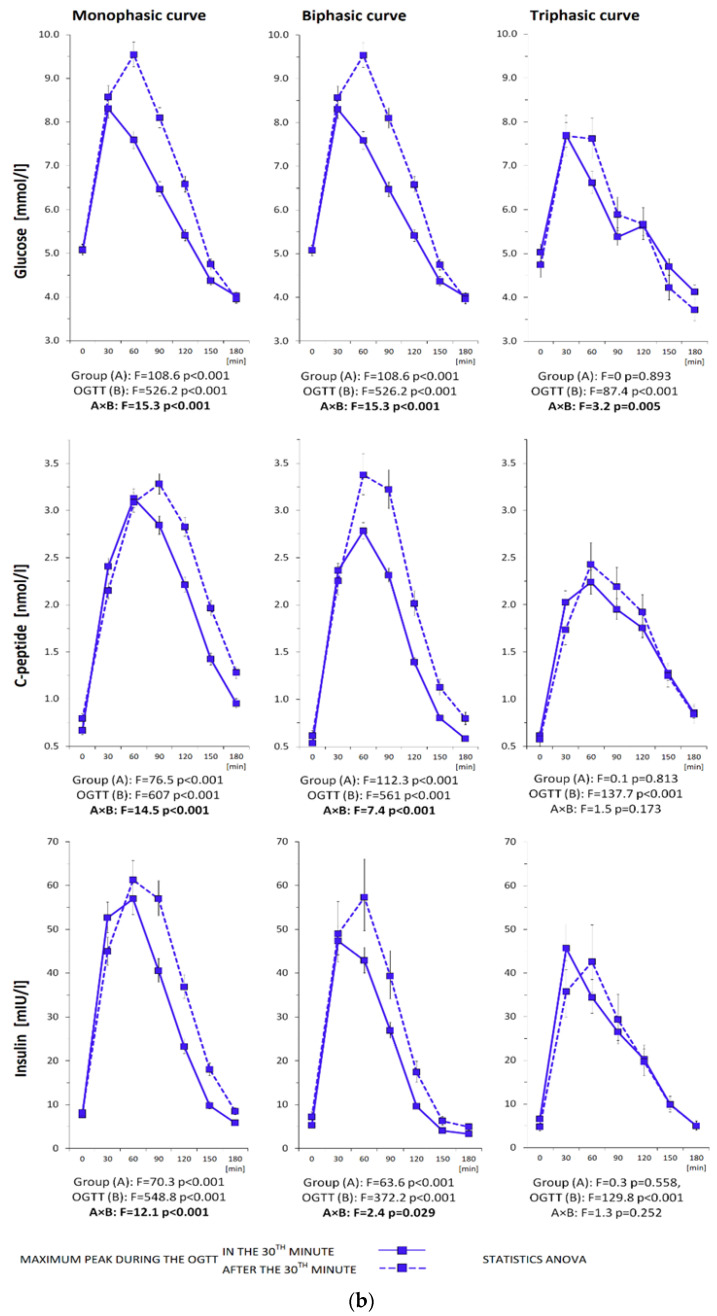
(**a**) The course of glycemic, insulinemic, and C-peptide curves during the 3 h OGTT demonstrating the shift of the peak from 30th min to the later phases of the test divided according to the type of trajectory in women. (**b**) The course of glycemic, insulinemic, and C-peptide curves during the 3 h OGTT demonstrating the shift of the peak from 30th min to the later phases of the test divided according to the type of trajectory in men.

Appropriate experimental calculations and data analysis methods for assessing both IS and beta cell function were used [23,24,25,26,27,28,32,33]. In case of skewed distribution and non-constant variance, variables were transformed by power transformations to reach data symmetry and homoscedasticity prior to further processing [34]. The homogeneity and distribution of the transformed data were checked by residual analysis, as described elsewhere [35]. A multiple comparison general linear model (GLM) ANOVA was used to examine blood glucose, insulin, and C-peptide levels during the OGTT between the curve type groups, with “group” and “OGTT” course as independent categorical factors A and B, respectively (Figures 1 and 3a,b) or with “sex” and “OGTT” course as independent categorical factors A and B, respectively (Figure 2). Parametric analysis using one-way ANOVA was then used to compare anthropometric and biochemical characteristics between the groups (Tables 1a–c, 5a–c and 6a–c). Bonferroni’s all-pairwise multiple comparison test was used to identify pairs of significantly different groups among all the groups tested (Tables 3a–c and 4a–c). Values of *p* < 0.05 (two samples) were considered significant. Chi2 statistics was used to test hypotheses about the distribution of the categorical data. The statistical software Statgraphics Centurion XVI 16.0.07, Statpoint Technologies, Inc., Warrenton, VA, USA, was used for testing.

## 3. Results

### 3.1. Metabolic Characterization of the Subjects Divided by Gender

A biochemically and anthropometrically characterized cohort of participants is reported in Table 1a–c. Data of the examined subjects are divided by gender.

When considering the presentation of health disorders (Table 2), 17 participants (1.3%) were newly diagnosed with T2DM. Impaired fasting glucose (IFG) was present in 100 individuals (8%), with impaired glucose tolerance (IGT) detected at the 120th min of the OGTT in 83 participants (6.6%). Impaired glucose regulation (IGR), including either IFG or IGT or both disorders, as well as overt DM2, was present in 167 individuals (13.2%). The remaining 1095 participants (86.8%) had normal glucose tolerance (NGT). On the other side, 139 subjects (11%) had metabolic syndrome (MS) according to the NCEP_ATPIII criteria [30]. Among women, 454 (43.9%) had positive histories of gestational diabetes mellitus (GDM), and 189 (18.3%) were diagnosed with polycystic ovary syndrome (PCOS) according to the European Society of Human Reproduction and Embryology consensus [31]. With regard to the glycemic curve shape, 70% of participants reached peak glucose concentrations at the 30th min of the OGTT, indicating that the maximum glucose concentrations shifted to the 60th min in 30% of the participants. Accordingly, the peak for the insulin levels shifted to the later stages of the OGTT (i.e., after the 30th min) in 57.7% of participants and 84% for C-peptide.

Although men and women did not differ in age, numerous metabolic gender differences were evident. In men, we observed an unfavorable profile in the lipid spectrum (lower HDL and higher LDL and triacylglycerols). In addition, systematic differences were present in the thyroid hormones and liver enzymes, showing higher levels (except TSH) in men, albeit with medians within a normal laboratory range (Table 1a). It is particularly important that men also differed from women in many parameters of glucose metabolism, especially in terms of lower glucose-induced insulin and C-peptide secretion (lower insulinemia from the 60th to 180th min, AUC ins, C-peptide from the 120th to 180th min, and AUC cp) (Table 1b). Therefore, men had also lower indices of pancreatic beta cell function (HOMA-beta, IGI, and AUC ins/AUC gluc ratio, as well as AUC cp/AUC gluc ratio), disposition indices, and the adaptation index. Hepatic extraction of insulin was higher in men (Table 1c). The course of glycemic, insulinemic, and C-peptide curves for women and men with a focus on the differences in individual times of the 3 h OGTT is shown in Figure 2. It clearly shows the higher and delayed curve of insulin secretion in women compared to men (especially in the biphasic and triphasic curves). The issue of the OGTT-derived trajectories, as well as the location of the peak of the secretory maximum, will be addressed in the following sections. Due to the systematic differences observed in the biochemical parameters between women and men, especially with regard to the differences in glucose homeostasis, we decided to maintain keeping the genders separate in the subsequent evaluation.

### 3.2. Metabolic Characterization of the Subjects Divided by the Shape of the Glycemic Curve

Among the total number of 1262 evaluated 3 h glycemic curves, 633 (50%) were monophasic, 221 (17.5%) biphasic, 351 (28%) triphasic, and 57 (4.5%) multiphasic. The vast majority of newly diagnosed diabetics (15 out of 17, which is 88.2%), as well as 70% of subjects with IFG and 78.3% with IGT, showed a monophasic shape. Therefore, overall, 75.4% of the people with a monophasic curve fell into the group with impaired glucose regulation. In addition, 74.8% of subjects with metabolic syndrome fell into the group with a monophasic curve. However, almost half of people (46.3%) with normal glucose tolerance also had a monophasic curve shape. As for women, the monophasic group included 53.3% with a history of GDM and 46% diagnosed with PCOS. Table 2 provides an overview of the diagnoses and metabolic characteristics of the participants divided according to the shape of the glycemic curves, including the specification of glucose, insulin, and C-peptide culmination.

Table 2 also demonstrates the distribution of women and men in the shape groups, and it is clear that it differed significantly. The most fundamental difference consisted in the distribution between the bi- and triphasic groups: a higher percentage of men had a biphasic curve (33% vs. 14% of women), while a higher percentage of women had a triphasic curve (30% vs. 19% of men) (*p* < 0.01). The percentage of women and men in the multiphasic group was similar, slightly exceeding 4%.

**Table 2:** Illustration of diagnoses and metabolic characteristics in groups divided by the shape of the glycemic curve.

	Total		Monophasic Curve		Biphasic Curve		Triphasic Curve		Multiphasic Curve	
	1262	%	633	%	221	%	351	%	57	%
WOMEN/MEN	1035/227	82.0/18.0	533/100	51.5/44.1	147/74	14.2/32.6	308/43	29.8/18.9	47/10	4.5/4.4
T2DM	17	1.3	15	88.2	1	5.9	1	5.9	0	0
GDM history	454	43.9	242	53.3	62	13.7	134	29.5	16	3.5
PCOS	189	18.3	87	46.0	24	12.7	64	33.9	14	7.4
IFG	100	8.0	70	70.0	8	8.0	21	21.0	1	1.0
IGT	83	6.6	65	78.3	2	2.4	15	18.1	1	1.2
IGR	167	13.2	126	75.4	11	6.6	29	17.4	1	0.6
NGT	1095	86.8	507	46.3	210	19.2	322	29.4	56	5.1
MS	139	11.0	104	74.8	10	7.2	24	17.3	1	0.7
PEAK in 30 min_GLUC	887	70.3	369	41.6	184	20.7	280	31.6	54	6.1
PEAK in 30 min_INS	534	42.3	195	36.5	105	19.7	192	36.0	42	7.9
PEAK in 30 min_CP	197	15.6	52	26.4	47	23.9	73	37.1	25	12.7

Data are given as absolute values and derived %. Explanatory notes and abbreviations for the tabulated parameters are available in Appendix A, Table A1.

As for women, the anthropometric and metabolic characterization of the shape groups is shown in Table 3a–c. The comparison is based on medians, which is appropriate given the skewed distribution of the vast majority of biological data. The monophasic group had higher waist circumference and higher systolic blood pressure compared to the bi-, tri-, and multiphasic groups. Regarding other components of metabolic syndrome, higher triacylglycerols were observed in monophasic women, although with medians and quartile ranges within the normal laboratory range. In addition, fasting glucose, insulin, and C-peptide levels, as well as AUC gluc, AUC ins, and AUC cp, were the largest in the monophasic group. Furthermore, higher basal insulin resistance (HOMA-R) and lower dynamic insulin sensitivity (QUICKI, OGIS 2 h, ISIcomp, MCRest, Si (oral), and PREDIM) were observed in this group. Additionally, some dynamic indices reflecting beta cell function (IGI, IGI cp, and IGI × ISIcomp) were also lower.

Differences observed in the women between the bi- and triphasic, bi- and multiphasic, or tri- and multiphasic groups were limited exclusively to glucose metabolism (Table 3a–c). Total stimulated AUC gluc levels were higher in triphasic women compared to bi- and multiphasic, although the beta cell function shown by IGI and IGI cp was higher in bi- and multiphasic women compared to triphasic. Therefore, IS was higher in biphasic and multiphasic women (according to OGIS 2 h and PREDIM, and Si(oral) indices, respectively) in comparison with the triphasic group. Thus, though not very common in women, the biphasic and multiphasic categories of glycemic curves present in 14.2 and 4.5%, respectively, appear more favorable for glucose homeostasis than the more common (29.8%) triphasic category.

As for men, the anthropometric and metabolic characterization of the shape groups is shown in Table 4a–c. Similar to women, the male monophasic group was characterized by higher blood pressure (systolic and diastolic) and waist circumference. Men belonging to the monophasic group also had a less favorable lipid spectrum, manifested by a higher total and LDL cholesterol, especially compared to the biphasic group, though again with medians within the normal laboratory range for all groups. Fasting glucose, insulin, and C-peptide levels, as well as AUC gluc, AUC ins, and AUC cp, were the largest in the monophasic group, similar to the situation in women, as displayed in Figure 2. Accordingly, higher HOMA-R and lower indices of IS (QUICKI, OGIS 2 h, ISIcomp, MCRest, Si(oral), and PREDIM) were observed in monophasic men. Additionally, IGI, IGI cp, and IGI × ISIcomp indices reflecting beta cell function were lower, which largely corresponds to the results observed in women.

Differences observed between the bi- and triphasic groups of men in anthropometry were limited to abdominal and waist circumferences and WHR (all these parameters were larger in triphasic compared to biphasic men). In accordance, higher total cholesterol was found in triphasic compared to biphasic men. Concerning glucose homeostasis, insulin sensitivity (OGIS 2 h, MCRest, and PREDIM) was correspondingly higher, and AUC gluc was lower in men showing a biphasic trajectory compared to those showing a triphasic one. In addition, four indices of beta cell function but not AUC ins or AUC cp were higher in biphasic compared to triphasic men. Thus, the biphasic curve, which is significantly more prevalent in men (32.6%), appears to be associated with some health benefits compared to the triphasic curve, which is considerably rarer among men (18.9%). Due to the low numerical prevalence (n = 10), it would not be appropriate to draw conclusions from the multiphasic curves in men, but it is worth noting the significantly higher levels of beta cell function in multiphasic men compared to triphasic (according to IGI simplified, IGI cp, IGI simplified cp, ΔAUC ins/ΔAUC gluc, and ΔAUC cp/ΔAUC gluc indices of beta cell function) and even compared to biphasic men (according to the IGI index).

**Table 3:** (**a**) Anthropometric and metabolic characterization of women divided by the shape of the glycemic curve. (**b**) OGTT descriptions of women divided by the shape of the glycemic curve. (**c**) Glucose homeostasis of women divided by the shape of the glycemic curve.

**(a)**											
**Women** **n = 1035 (100%)**	**Monophasic Curve** **n = 533 (51.5%)**	**Biphasic Curve** **n = 147 (14.2%)**	**Triphasic Curve** **n = 308 (29.8%)**	**Multiphasic Curve** **n = 47 (4.5%)**	***p*** **-Level**	**Mono × Bi**	**Mono × Tri**	**Mono × Multi**	**Bi × Tri**	**Bi × Multi**	**Tri** × **Multi**
Anthropometric Parameters											
Age [years]	33.8 (28, 41)	32.1 (25.7, 38.9)	33.6 (28.1, 38.6)	29.3 (25.2, 34)	<0.001	*		*		*	*
BMI [kg/m^2^]	24.4 (21, 28.8)	22.8 (20.3, 27.6)	23 (20.7, 26)	23 (20.8, 25.4)	<0.001	*	*	*			
BAI [%]	28.8 (25.6, 32.6)	26.8 (24.7, 30.7)	27.6 (25.4, 30.8)	27.5 (25.6, 29.5)	<0.001	*	*	*			
Systolic blood pressure [mmHg]	115 (106, 124)	111 (103, 122)	111 (103, 120)	110 (103, 122)	0.005	*	*				
Diastolic blood pressure [mmHg]	72.5 (66, 80)	70 (64.5, 77.5)	71 (65, 78)	71 (65.3, 76)	0.05	*	*				
Abdominal circumference [cm]	87.5 (79.1, 97.3)	83 (77, 95.8)	84 (78.1, 91.7)	82.8 (79.3, 87.6)	0.001	*	*	*			
Hip circumference [cm]	101 (95, 109)	99.7 (94.5, 105)	99.4 (94, 106)	99.5 (94.9, 104)	0.004		*				
Waist circumference [cm]	78 (70.3, 88)	73.2 (69.5, 84.9)	74 (69.5, 80.8)	73.8 (70.5, 81.3)	<0.001	*	*	*			
WHR	0.775 (0.731, 0.821)	0.756 (0.718, 0.8)	0.754 (0.722, 0.794)	0.744 (0.714, 0.795)	<0.001	*	*	*			
Biochemical Parameters											
Total cholesterol [mmol/L]	4.64 (4.09, 5.26)	4.49 (4.05, 5.08)	4.55 (3.98, 5.12)	4.33 (3.92, 4.98)	0.069			*			
HDL cholesterol [mmol/L]	1.54 (1.32, 1.79)	1.58 (1.34, 1.88)	1.6 (1.35, 1.86)	1.61 (1.35, 1.89)	0.348						
LDL cholesterol [mmol/L]	2.62 (2.1, 3.12)	2.44 (2.07, 2.93)	2.45 (2.07, 3)	2.42 (2.02, 2.77)	0.175						
Triacylglycerols [mmol/L]	0.9 (0.65, 1.27)	0.75 (0.582, 1.15)	0.78 (0.56, 1.06)	0.73 (0.54, 1.05)	<0.001	*	*	*			
Urea [mmol/L]	4.2 (3.6, 5)	4.21 (3.6, 4.9)	4.26 (3.61, 5.01)	4.1 (3.6, 4.9)	0.666						
Uric acid [umol/L]	258 (226, 293)	259 (212, 292)	248 (215, 290)	237 (216, 271)	0.047		*	*			
Creatinine [umol/L]	65 (59, 72.7)	65 (59, 71)	64 (58, 70.1)	65 (57.6, 72.3)	0.439						
TSH [mIU/L]	2.25 (1.56, 3.29)	2.36 (1.72, 3.28)	2.39 (1.63, 3.09)	2.29 (1.71, 3.25)	0.798						
Free T4 [pmol/L]	15.1 (13.8, 16.5)	15 (13.6, 16.5)	14.9 (13.5, 16.6)	14.9 (13.8, 16.7)	0.922						
Free T3 [pmol/L]	4.84 (4.4, 5.33)	4.77 (4.48, 5.48)	4.75 (4.32, 5.22)	4.91 (4.51, 5.41)	0.368						
ALT [ukat/L]	0.3 (0.23, 0.395)	0.27 (0.23, 0.34)	0.28 (0.225, 0.35)	0.29 (0.23, 0.35)	0.098		*				
AST [ukat/L]	0.34 (0.29, 0.42)	0.33 (0.29, 0.4)	0.33 (0.28, 0.39)	0.34 (0.31, 0.422)	0.18						
GGT [ukat/L]	0.23 (0.17, 0.34)	0.21 (0.17, 0.287)	0.21 (0.16, 0.27)	0.19 (0.15, 0.298)	0.012		*				
**(b) **											
**Women** **n = 1035 (100%)**	**Monophasic Curve** **n = 533 (51.5%)**	**Biphasic Curve** **n = 147 (14.2%)**	**Triphasic Curve** **n = 308 (29.8%)**	**Multiphasic Curve** **n = 47 (4.5%)**	***p*** **-Level**	**Mono × Bi**	**Mono × Tri**	**Mono × Multi**	**Bi × Tri**	**Bi × Multi**	**Tri** × **Multi**
Ogtt Descriptions											
Glucose 0 min [mmol/L]	4.8 (4.5, 5.1)	4.7 (4.5, 5)	4.7 (4.4, 5)	4.5 (4.35, 5)	0.002		*	*			
Glucose 30 min	8 (7.1, 8.9)	7.6 (6.9, 8.5)	7.2 (6.3, 8)	6.7 (6.05, 7.35)	<0.001	*	*	*	*	*	
Glucose 60 min	7.9 (6.7, 9.4)	6.6 (5.65, 7.85)	6.2 (5.1, 7.1)	4.9 (4.4, 5.85)	<0.001	*	*	*	*	*	*
Glucose 90 min	6.7 (5.7, 8.2)	5.5 (4.5, 6.1)	5.1 (4.5, 5.9)	4.6 (4.05, 5.25)	<0.001	*	*	*		*	*
Glucose 120 min	5.8 (5, 6.9)	4.3 (3.6, 5.15)	5.6 (4.9, 6.4)	5 (4.25, 5.45)	<0.001	*	*	*	*	*	*
Glucose 150 min	4.6 (3.9, 5.6)	3.3 (2.8, 4.35)	4.9 (4.2, 5.6)	4 (3.3, 4.55)	<0.001	*	*	*	*	*	*
Glucose 180 min	3.8 (3.3, 4.4)	3.9 (3.6, 4.5)	4 (3.4, 4.6)	4.1 (3.6, 4.95)	<0.001	*	*	*			
C-peptide 0 min [nmol/L]	0.61 (0.49, 0.81)	0.58 (0.475, 0.74)	0.58 (0.47, 0.72)	0.56 (0.465, 0.685)	0.013	*	*	*			
C-peptide 30 min	2.05 (1.64, 2.59)	2.1 (1.76, 2.71)	2.03 (1.68, 2.46)	2.07 (1.59, 2.72)	0.236				*		
C-peptide 60 min	2.86 (2.29, 3.6)	2.81 (2.44, 3.38)	2.44 (2.06, 2.99)	2.1 (1.69, 2.84)	<0.001		*	*	*	*	*
C-peptide 90 min	2.87 (2.3, 3.6)	2.57 (2.16, 3.1)	2.24 (1.82, 2.63)	2 (1.55, 2.58)	<0.001	*	*	*	*	*	*
C-peptide 120 min	2.49 (1.96, 3.28)	1.82 (1.44, 2.26)	2.13 (1.75, 2.6)	1.81 (1.41, 2.21)	<0.001	*	*	*	*		*
C-peptide 150 min	1.79 (1.35, 2.4)	1.11 (0.83, 1.58)	1.68 (1.36, 2.12)	1.16 (0.89, 1.59)	<0.001	*	*	*	*		*
C-peptide 180 min	1.13 (0.87, 1.58)	0.79 (0.605, 1.19)	1.08 (0.84, 1.37)	0.9 (0.59, 1.29)	<0.001	*	*	*	*		*
Insulin 0 min [mIU/L]	6.4 (4.5, 10)	6 (4.25, 8.8)	5.9 (4.3, 8.33)	5.4 (4.3, 7.5)	0.031		*	*			
Insulin 30 min	45.8 (31.8, 70.9)	50.2 (35.3, 67.7)	49 (33.9, 67.1)	54.7 (35.6, 77.3)	0.6						
Insulin 60 min	56.6 (38.4, 84.2)	55.2 (39.6, 73.9)	44.9 (32.2, 62.2)	34 (26.7, 52.6)	<0.001		*	*	*	*	*
Insulin 90 min	47.7 (31.4, 74.2)	38.1 (27.6, 53.8)	33 (22.6, 46.4)	32.5 (19.5, 40.7)	<0.001	*	*	*	*	*	
Insulin 120 min	31.4 (20.3, 53.6)	19.1 (10.8, 28.1)	29.4 (21.2, 42)	20.6 (14, 35.3)	<0.001	*	*	*	*		*
Insulin 150 min	15.5 (8.4, 30)	6.9 (4.05, 14)	17.2 (10.6, 27.6)	8.7 (4.7, 18.4)	<0.001	*		*	*		*
Insulin 180 min	6.8 (4.4, 14.6)	5.1 (3.1, 10.7)	6.4 (4.1, 10.7)	5.9 (3.35, 10.4)	<0.001	*	*		*		
AUC gluc 30 min	191 (176, 209)	186 (171, 202)	179 (162, 194)	170 (158, 182)	<0.001	*	*	*	*	*	
AUC gluc	1130 (1010, 1290)	965 (869, 1060)	999 (898, 1110)	902 (801, 972)	<0.001	*	*	*	*	*	*
ΔAUC gluc	270 (156, 398)	158 (55.5, 239)	156 (84.4, 261)	81.8 (42, 155)	<0.001	*	*	*		*	*
AUC ins 30 min	4810 (3320, 7310)	5210 (3780, 6890)	4920 (3580, 6750)	5590 (3640, 7900)	0.731						
AUC ins	38,100 (27,100, 57,300)	34,100 (25,900, 43,100)	32,900 (24,900, 44,600)	27,200 (20,400, 41,600)	<0.001	*	*	*			
ΔAUC ins	30,200 (21,500, 47,300)	27,000 (19,800, 33,500)	25,900 (19,600, 36,200)	22,000 (15,700, 34,000)	<0.001	*	*	*			
AUC cp 30 min	40,200 (32,600, 50,900)	41,100 (34,400, 51,500)	39,200 (32,800, 48,000)	39,500 (31,300, 50,500)	0.234						
AUC cp	393 × 10^3^ (323, 487) × 10^3^	343 × 10^3^ (300, 401) × 10^3^	344 × 10^3^ (293, 411) × 10^3^	305 × 10^3^ (244, 403) × 10^3^	<0.001	*	*	*		*	*
ΔAUC cp	275 × 10^3^ (228, 346) × 10^3^	237 × 10^3^ (195, 275) × 10^3^	237 × 10^3^ (198, 287) × 10^3^	194 × 10^3^ (156, 266) × 10^3^	<0.001	*	*	*		*	*
**(c) **											
**Women** **n = 1035 (100%)**	**Monophasic Curve** **n = 533 (51.5%)**	**Biphasic Curve** **n = 147 (14.2%)**	**Triphasic Curve** **n = 308 (29.8%)**	**Multiphasic Curve** **n = 47 (4.5%)**	***p*** **-Level**	**Mono × Bi**	**Mono × Tri**	**Mono × Multi**	**Bi × Tri**	**Bi × Multi**	**Tri** × **Multi**
Insulin Sensitivity/Resistance											
HOMA-R	1.36 (0.915, 2.22)	1.27 (0.905, 1.88)	1.24 (0.837, 1.8)	1.09 (0.86, 1.52)	0.005		*	*			
QUICKI	0.365 (0.338, 0.389)	0.369 (0.347, 0.39)	0.37 (0.349, 0.395)	0.378 (0.358, 0.393)	0.01		*	*			
OGIS 2h	449 (397, 490)	481 (451, 514)	461 (426, 495)	477 (439, 517)	<0.001	*	*	*	*		
OGIS 3h	502 (443, 549)	516 (451, 556)	504 (465, 548)	490 (454, 561)	0.138	*					
ISIcomp	7.5 (4.56, 10.9)	8.89 (6.49, 11.3)	8.71 (6.15, 11.7)	10.5 (7.24, 13.3)	<0.001	*	*	*			
MCRest	9.42 (7.55, 10.6)	10.5 (9.37, 11.3)	10.1 (9.01, 11)	10.6 (9.13, 11.4)	<0.001	*	*	*			
Si(oral)	0.127 (0.059, 0.202)	0.197 (0.127, 0.301)	0.175 (0.109, 0.282)	0.268 (0.129, 0.401)	<0.001	*	*	*			*
PREDIM	6.63 (4.84, 8.51)	7.87 (6.4, 9.91)	6.93 (5.73, 8.68)	7.58 (6.11, 9.09)	<0.001	*	*	*	*		
Beta cell function											
HOMA-beta	104 (72, 165)	100 (73, 159)	105 (76.4, 146)	101 (70.9, 147)	0.995						
Ins0/Gluc0	7.87 (5.76, 12.2)	7.58 (5.51, 11.3)	7.55 (5.84, 10.2)	7.36 (5.6, 10.4)	0.124		*				
Cp0/Gluc0	434 (318, 675)	418 (304, 624)	417 (322, 563)	406 (309, 575)	0.125						
IGI	76.5 (50.2, 129)	94 (59.4, 157)	114 (74.7, 164)	131 (84.4, 222)	<0.001	*	*	*	*	*	
IGI simplified	34.7 (24.1, 52.9)	40.2 (28.4, 56.5)	41.2 (30.8, 55.4)	46.4 (32.5, 69.4)	<0.001	*	*	*			
IGI cp	453 (324, 657)	559 (373, 815)	605 (445, 822)	700 (539, 1110)	<0.001	*	*	*	*	*	
IGI simplified cp	1910 (1330, 2920)	2220 (1570, 3110)	2270 (1700, 3060)	2560 (1790, 3830)	<0.001	*	*	*			
AUC ins/AUC gluc	33.4 (24.8, 50)	35.6 (26.4, 44.6)	33.3 (26.4, 43.9)	32.1 (23.1, 46.2)	0.62						
ΔAUC ins/ΔAUC gluc	129 (78.4, 235)	193 (113, 505)	186 (113, 299)	319 (159, 544)	<0.001	*	*	*			*
AUC cp/AUC gluc	353 (283, 434)	349 (304, 448)	351 (293, 403)	337 (273, 418)	0.419						
ΔAUC cp/ΔAUC gluc	1060 (723, 1790)	1690 (1090, 3740)	1580 (996, 2620)	2360 (1560, 4180)	<0.001	*	*	*			*
Disposition Indices											
IGI × ISIcomp	244 (194, 304)	297 (239, 377)	286 (234, 354)	317 (258, 401)	<0.001	*	*	*		*	*
OGIS 3h × AUCins	19.0 × 10^6^ (14.2, 27.2) × 10^6^	17.2 × 10^6^ (13.4, 21.1) × 10^6^	16.7 × 10^6^ (13.3, 22.5) × 10^6^	14.0 × 10^6^ (10.7, 22.1) × 10^6^	<0.001	*	*	*			
Adaptation Index											
OGIS 3h × AUCcp	19.2 × 10^7^ (16.6, 23.0) × 10^7^	17.5 × 10^7^ (14.5, 20.7) × 10^7^	17.5 × 10^7^ (14.9, 20.5) × 10^7^	15.8 × 10^7^ (11.9, 18.9) × 10^7^	<0.001	*	*	*		*	*
Hepatic Extraction											
HE	67.5 (59.6, 73.4)	67.7 (61.8, 73.7)	68 (61.9, 72.6)	68.4 (59, 73.6)	0.307						

Data are given as median (95% LCL; 95% UCL), *p*-levels according to ANOVA test, and * *p*-level < 0.05 according to Bonferroni’s all-pairwise multiple comparison test. Explanatory notes and abbreviations for the tabulated parameters are available in Appendix A, Table A1.

**Table 4:** (**a**) Anthropometric and metabolic characterization of men divided by the shape of the glycemic curve. (**b**) OGTT descriptions of men divided by the shape of the glycemic curve. (**c**) Glucose homeostasis of men divided by the shape of the glycemic curve.

**(a)**											
**Men** **n = 227 (100%)**	**Monophasic Curve** **n = 100 (44.1%)**	**Biphasic Curve** **n = 74 (32.6%)**	**Triphasic Curve** **n = 43 (18.9%)**	**Multiphasic Curve** **n = 10 (4.4%)**	***p*-Level**	**Mono × Bi**	**Mono × Tri**	**Mono × Multi**	**Bi × Tri**	**Bi × Multi**	**Tri × Multi**
NGT/IFG + IGT/T2DM	73/22/5	72/2/0	36/7/0	10/0/0							
Anthropometric Parameters											
Age [years]	38.4 (26.7, 49.3)	27.2 (23.7, 37.6)	38 (30.8, 47.2)	26.7 (25.9, 29.1)	<0.001	*		*	*		*
BMI [kg/m^2^]	25.6 (23.2, 29.8)	24.1 (21.8, 26.4)	25.2 (23.4, 27.5)	25.6 (23.2, 28.6)	0.004	*					
BAI [%]	24 (21.9, 26.3)	22.3 (20.7, 23.8)	23.2 (21.1, 24.6)	23.6 (20.8, 25.4)	0.001	*					
Systolic blood pressure [mmHg]	128 (116, 140)	120 (113, 129)	122 (113, 132)	131 (119, 138)	0.008	*	*				
Diastolic blood pressure [mmHg]	77 (70, 86.3)	73 (67.8, 77.3)	72 (64.5, 80)	71.5 (61.3, 82)	0.001	*	*	*			
Abdominal circumference [cm]	91.7 (85.3, 105)	85.8 (79.4, 93)	91 (85.7, 96.3)	93.3 (87.6, 96.8)	0.001	*			*		
Hip circumference [cm]	100 (96.1, 107)	98.3 (95, 102)	100 (95.5, 105)	103 (96.5, 106)	0.049	*					
Waist circumference [cm]	88 (80.7, 102)	82.2 (76.9, 89)	88 (82, 93.6)	90.3 (82, 95.6)	<0.001	*			*		
WHR	0.873 (0.832, 0.952)	0.841 (0.802, 0.887)	0.88 (0.84, 0.926)	0.867 (0.804, 0.919)	<0.001	*			*		
Biochemical Parameters											
Total cholesterol [mmol/L]	4.82 (4.31, 5.41)	4.49 (3.66, 5.01)	4.85 (4.15, 5.38)	4.02 (3.67, 4.58)	0.013	*			*		
HDL cholesterol [mmol/L]	1.26 (1.03, 1.51)	1.26 (1.04, 1.49)	1.23 (1.09, 1.51)	1.31 (1.05, 1.58)	0.799						
LDL cholesterol [mmol/L]	2.92 (2.31, 3.42)	2.61 (2.05, 3.02)	2.88 (2.35, 3.45)	2.15 (1.74, 2.62)	0.037	*		*			
Triacylglycerols [mmol/L]	1.12 (0.767, 1.7)	1.01 (0.715, 1.42)	0.98 (0.765, 1.62)	0.83 (0.672, 1.11)	0.362						
Urea [mmol/L]	4.95 (4.1, 5.8)	4.9 (4.2, 6)	5.2 (4.51, 5.85)	4.63 (3.65, 4.85)	0.304						
Uric acid [umol/L]	341 (294, 400)	329 (297, 362)	308 (282, 359)	362 (322, 388)	0.024		*				
Creatinine [umol/L]	80 (73.9, 89)	81.9 (74.2, 88.4)	79 (71, 89)	81.9 (73.5, 96.5)	0.889						
TSH [mIU/L]	2.17 (1.46, 2.75)	2.07 (1.53, 2.96)	1.82 (1.31, 2.62)	2.46 (2.11, 2.83)	0.343						
Free T4 [pmol/L]	15.8 (14.3, 17.2)	15.8 (14.4, 17.3)	15.6 (14.6, 17.6)	16.9 (16, 18)	0.358						
Free T3 [pmol/L]	5.15 (4.7, 5.67)	5.41 (4.97, 5.94)	5.19 (4.92, 5.57)	5.11 (4.93, 5.66)	0.014	*					
ALT [ukat/L]	0.435 (0.34, 0.55)	0.41 (0.313, 0.605)	0.37 (0.29, 0.51)	0.345 (0.31, 0.447)	0.852						
AST [ukat/L]	0.405 (0.363, 0.48)	0.435 (0.358, 0.512)	0.385 (0.358, 0.502)	0.385 (0.357, 0.412)	0.979						
GGT [ukat/L]	0.4 (0.285, 0.57)	0.33 (0.22, 0.44)	0.37 (0.23, 0.585)	0.23 (0.21, 0.315)	0.035	*					
**(b)**											
**Men** **n = 227 (100%)**	**Monophasic Curve** **n = 100 (44.1%)**	**Biphasic Curve** **n = 74 (32.6%)**	**Triphasic Curve** **n = 43 (18.9%)**	**Multiphasic Curve** **n = 10 (4.4%)**	***p*-Level**	**Mono × Bi**	**Mono × Tri**	**Mono × Multi**	**Bi × Tri**	**Bi × Multi**	**Tri × Multi**
Ogtt Descriptions											
Glucose 0 min [mmol/L]	5 (4.7, 5.43)	4.7 (4.43, 5.07)	4.9 (4.7, 5.3)	5 (4.55, 5.17)	<0.001	*			*		
Glucose 30 min	8.5 (7.5, 9.4)	7.85 (6.88, 8.7)	7.8 (7, 8.5)	6.9 (6.72, 7.3)	<0.001	*	*	*			
Glucose 60 min	8.45 (7.4, 9.97)	6.65 (5.3, 8)	6.8 (5.9, 7.65)	4.75 (4.43, 5.3)	<0.001	*	*	*		*	*
Glucose 90 min	7.15 (6, 8.63)	4.85 (4.2, 5.98)	5.3 (4.8, 6.3)	4.6 (3.72, 5.23)	<0.001	*	*	*			*
Glucose 120 min	5.8 (5, 6.9)	3.8 (3.4, 4.5)	5.5 (4.75, 6.25)	4.55 (4.15, 4.9)	<0.001	*		*	*	*	*
Glucose 150 min	4.4 (3.9, 5.2)	3.3 (3, 3.57)	4.5 (4, 5.15)	3.3 (3.23, 3.55)	<0.001	*		*	*		*
Glucose 180 min	3.85 (3.5, 4.6)	4 (3.6, 4.2)	4 (3.6, 4.45)	3.8 (3.62, 4.1)	0.992						
C-peptide 0 min [nmol/L]	0.64 (0.48, 0.995)	0.52 (0.403, 0.663)	0.57 (0.465, 0.725)	0.63 (0.522, 0.708)	0.007	*					
C-peptide 30 min	2.14 (1.74, 2.76)	2.41 (1.9, 2.83)	1.91 (1.47, 2.48)	2.07 (1.86, 3.56)	0.001		*		*		*
C-peptide 60 min	3.04 (2.48, 3.87)	2.78 (2.38, 3.65)	2.25 (1.7, 2.91)	2.45 (2.13, 2.66)	<0.001		*	*	*		
C-peptide 90 min	3.05 (2.35, 3.94)	2.44 (1.83, 3.2)	2.01 (1.61, 2.6)	1.89 (1.72, 2.61)	<0.001	*	*	*	*		
C-peptide 120 min	2.51 (1.75, 3.34)	1.5 (1.04, 2.01)	1.81 (1.29, 2.56)	1.75 (1.64, 1.91)	<0.001	*	*		*		
C-peptide 150 min	1.54 (1.15, 2.33)	0.835 (0.692, 1.1)	1.26 (0.805, 1.76)	0.93 (0.823, 0.95)	<0.001	*	*	*	*		*
C-peptide 180 min	0.975 (0.72, 1.51)	0.59 (0.49, 0.738)	0.79 (0.59, 1.11)	0.675 (0.603, 0.733)	<0.001	*	*	*	*		
Insulin 0 min [mIU/L]	6.95 (5, 10.8)	5.55 (3.9, 7.75)	5.6 (4.25, 8.15)	6.8 (3.77, 7.07)	0.024	*	*				
Insulin 30 min	47 (33.2, 64.2)	47 (36.9, 62.8)	39.6 (27, 69.8)	49 (37.7, 87.2)	0.148						*
Insulin 60 min	58.4 (40.4, 89.3)	44.6 (29, 65.1)	38 (23.4, 62.8)	32 (28.3, 51.3)	<0.001	*	*	*			
Insulin 90 min	48.4 (28.4, 72.4)	25.6 (17.3, 43.7)	25.7 (17.1, 43)	26.6 (17.5, 35.5)	<0.001	*	*	*			
Insulin 120 min	27 (14.2, 52.6)	12.3 (6.43, 17.2)	19.9 (10.5, 39.8)	17.7 (15.7, 21.9)	<0.001	*			*	*	
Insulin 150 min	10.8 (5.83, 25.9)	4.55 (2.93, 7.15)	8.5 (5.05, 19.9)	4.55 (3.95, 6.38)	<0.001	*		*	*		*
Insulin 180 min	6.1 (3.2, 12.3)	3.35 (2.62, 5)	4.1 (2.4, 6.5)	3.8 (2.65, 5.6)	<0.001	*	*	*			
AUC gluc 30 min	203 (185, 224)	185 (174, 206)	192 (179, 202)	179 (170, 181)	<0.001	*	*	*			
AUC gluc	1160 (1050, 1310)	929 (838, 1040)	1030 (948, 1110)	849 (809, 921)	<0.001	*	*	*	*		*
ΔAUC gluc	273 (167, 372)	116 (54, 179)	170 (93, 245)	40.5 (33, 52.5)	<0.001	*	*	*	*		*
AUC ins 30 min	5050 (3490, 6770)	4670 (3680, 6410)	4120 (2720, 6930)	4930 (4030, 8320)	0.147		*				
AUC ins	36,300 (25,700, 56,900)	25,800 (18,800, 35,000)	29,300 (17,700, 43,200)	27,500 (21,300, 32,800)	<0.001	*	*				
ΔAUC ins	29,100 (19,400, 43,200)	19,400 (13,500, 26,700)	20,500 (12,300, 33,700)	21,400 (16,300, 26,900)	<0.001	*	*				
AUC cp 30 min	43,100 (34,400, 56,400)	45,000 (35,600, 52,400)	36,500 (29,000, 47,600)	41,000 (36,300, 66,000)	0.034		*		*		
AUC cp	405 × 10^3^ (303, 517) × 10^3^	314 × 10^3^ (264, 403) × 10^3^	305 × 10^3^ (237, 402) × 10^3^	289 × 10^3^(275, 372) × 10^3^	<0.001	*	*	*			
ΔAUC cp	269 × 10^3^ (208, 351) × 10^3^	219 × 10^3^ (172, 277) × 10^3^	203 × 10^3^ (148, 261) × 10^3^	183 × 10^3^ (169, 235) × 10^3^	<0.001	*	*	*			
**(c)**											
**Men** **n = 227 (100%)**	**Monophasic Curve** **n = 100 (44.1%)**	**Biphasic Curve** **n = 74 (32.6%)**	**Triphasic Curve** **n = 43 (18.9%)**	**Multiphasic Curve** **n = 10 (4.4%)**	***p*-Level**	**Mono × Bi**	**Mono × Tri**	**Mono × Multi**	**Bi × Tri**	**Bi × Multi**	**Tri × Multi**
Insulin Sensitivity/Resistance											
HOMA-R	1.52 (1.02, 2.66)	1.15 (0.77, 1.7)	1.3 (0.894, 1.74)	1.44 (0.786, 1.6)	0.002	*	*				
QUICKI	0.359 (0.33, 0.382)	0.375 (0.352, 0.401)	0.368 (0.351, 0.391)	0.361 (0.356, 0.4)	0.004	*	*				
OGIS 2h	433 (382, 477)	496 (461, 525)	442 (410, 471)	455 (416, 484)	<0.001	*			*	*	
OGIS 3h	477 (407, 529)	497 (475, 532)	472 (444, 515)	484 (459, 511)	0.056	*					
ISIcomp	7.7 (4.13, 10.7)	10.5 (8.2, 14.3)	9.22 (6.31, 14.2)	9.7 (8.04, 13.3)	<0.001	*	*				
MCRest	9.24 (7.25, 10.2)	10.6 (9.89, 11.2)	9.84 (9.02, 10.6)	9.98 (8.75, 10.8)	<0.001	*	*	*	*		
Si(oral)	0.098 (0.046, 0.168)	0.206 (0.134, 0.365)	0.179 (0.094, 0.327)	0.189 (0.159, 0.295)	<0.001	*	*	*			
PREDIM	6.14 (4.36, 7.56)	8.16 (6.95, 10.2)	6.9 (5.81, 8.1)	6.46 (5.62, 8.62)	<0.001	*			*		
Beta Cell Function											
HOMA-beta	105 (69.4, 142)	103 (62.7, 146)	85 (57.6, 133)	101 (74.5, 128)	0.635						
Ins0/Gluc0	8.39 (6.13, 13.3)	7.3 (4.98, 9.83)	7.38 (4.85, 10.1)	7.78 (5.39, 9.27)	0.076	*					
Cp0/Gluc0	463 (338, 735)	403 (275, 542)	407 (267, 557)	429 (298, 511)	0.076	*					
IGI	72.7 (49.1, 108)	84 (56.3, 128)	80.9 (49.3, 142)	139 (83, 201)	0.008			*		*	
IGI simplified	34 (23.2, 48.8)	37.3 (26, 48.9)	28.6 (21.6, 51.2)	46.5 (31.4, 76.8)	0.117			*			*
IGI cp	471 (326, 650)	600 (420, 903)	459 (328, 631)	784 (654, 1400)	<0.001	*		*	*		*
IGI simplified cp	1880 (1280, 2690)	2060 (1430, 2700)	1580 (1190, 2820)	2560 (1730, 4240)	0.117			*			*
AUC ins/AUC gluc	32 (21.4, 46.6)	27.3 (22.1, 36)	27.6 (16.9, 37.4)	33.4 (25.3, 39.8)	0.125		*				
ΔAUC ins/ΔAUC gluc	129 (72.1, 212)	183 (112, 399)	154 (82.6, 256)	466 (310, 538)	<0.001	*		*	*		*
AUC cp/AUC gluc	343 (276, 418)	347 (290, 413)	296 (242, 345)	339 (324, 407)	0.011		*		*		
ΔAUC cp/ΔAUC gluc	1050 (744, 1680)	2010 (1420, 4240)	1310 (890, 1910)	4110 (2760, 6990)	<0.001	*		*	*		*
Disposition Indices											
IGI × ISIcomp	213 (167, 260)	302 (258, 351)	247 (206, 298)	346 (264, 422)	<0.001	*	*	*	*		*
OGIS 3h × AUCins	17.1 × 10^6^ (12.4, 24.5) × 10^6^	13.0 × 10^6^ (9.7, 16.0) × 10^6^	14.0 × 10^6^ (8.2, 19.6) × 10^6^	13.0 × 10^6^ (10.8, 16.4) × 10^6^	<0.001	*	*				
Adaptation Index											
OGIS 3h × AUCcp	18.8 × 10^7^ (15.6, 22.0) × 10^7^	15.8 × 10^7^ (13.1, 19.4) × 10^7^	15.0 × 10^7^ (12.5, 17.9) × 10^7^	13.9 × 10^7^ (13.4, 15.8) × 10^7^	<0.001	*	*	*			
Hepatic Extraction											
HE	68.3 (60.9, 75.9)	71.3 (66.6, 78.4)	69.7 (63.3, 77.3)	68.9 (62.4, 75)	0.074	*					

Data are given as median (95% LCL; 95% UCL), *p*-levels according to ANOVA test, and * *p*-level < 0.05 according to Bonferroni’s all-pairwise multiple comparison test. Explanatory notes and abbreviations for the tabulated parameters are available in Appendix A, Table A1.

### 3.3. Metabolic Characterization of the Subjects Divided by the Location of the Glycemic Peak

The shape of the glycemic curves, together with insulin and C-peptide curves demonstrating the shift of the peak from the 30th min of the OGTT to the later phases of the test, divided according to the shape type, are reported in women and men (Figure 3a and Figure 3b, respectively).

#### 3.3.1. Monophasic Curves

Within the monophasic curves, which proved to be less healthy for both sexes, the shift of the peak from the 30th min to the later phases was frequent (42%) and linked to further deterioration of glucose tolerance and components of MS. The worsening was evident in both fasting and stimulated blood glucose, insulinemia, C-peptide, indices of insulin sensitivity, and some indices of beta cell function, both in women and men (see Table 5a–c and Table 6a–c).

Regarding the monophasic glycemic curves in women, a more robust stature (larger waist circumference, abdominal circumference, WHR, and % of body fat according to BAI, as well as higher BMI) observed in the group with a delayed peak was reflected in deteriorated triacylglycerols and higher levels of uric acid and liver enzymes, although with medians within the normal laboratory range. Systematic differences were also found in glucose metabolism. Fasting glucose, insulin, and C-peptide levels, as well as AUC gluc, AUC ins, and AUC cp, were higher in monophasic women with a delayed peak. Furthermore, peak delay was associated with a worsening of insulin sensitivity, thus with higher requirements for insulin secretion, reflected in the indices of beta cell function (Figure 3a). A decrease in hepatic insulin extraction was also significant, which may be related to the observed higher levels of liver enzymes. In addition, considering the higher proportion of fat mass, this may indicate a worse condition of hepatocytes. The compared groups did not differ in age, which is important in the case of the above differences.

Concerning monophasic curves with a delayed peak in men, differences in anthropometry were not evident, and the main findings were higher stimulated glycemia accompanied, starting at the 90th min of the OGTT, by higher insulin and C-peptide levels. Hence, as also evidenced by MCRest and Si (oral) indices, a delayed peak in monophasic men was associated with lower insulin sensitivity.

#### 3.3.2. Biphasic Curves

Within biphasic curves, the delay of the maximum peak was much rarer (17%) compared to the monophasic. Furthermore, deterioration of the metabolic parameters associated with delayed culmination was less systematic than was the case with monophasic curves, with significant differences between women and men; similar observations in both genders were limited to stimulated blood glucose and C-peptide levels (AUC gluc and AUC cp) and the Si (oral) index of insulin sensitivity (see Table 5b,c and Table 6b,c).

#### 3.3.3. Triphasic Curves

Concerning the triphasic curves, the peak was delayed in 20%, similar to the biphasic ones. In women, this delay was associated with slightly larger waist circumference, abdominal circumference, and WHR, without projection into the lipid spectrum. Stimulated glycemia was higher, starting from the 60th min, which translated into a higher AUC gluc. Insulin sensitivity (OGIS 2 h, OGIS 3h, MCRest, Si (oral), and PREDIM) was lower, and indices of beta cell function were systematically lower in these women, together with the disposition and adaptation indices.

As for men, the shift of the maximum peak to the 60th min was not significantly associated with either anthropometric or metabolic parameters, which, however, may be due to the low prevalence of men in the triphasic group of glycemic curves.

#### 3.3.4. Multiphasic Curves

As regards multiphasic curves, peak delay occurred in 5% and was not accompanied by any changes in biochemical parameters. However, due to the low prevalence (peak delay was seen in only 3 out of 57 individuals of the multiphasic group, counting both men and women), we refrained from commenting on this aspect.

In summary, it can be assumed that, from a clinical point of view, a delay in a glycemic peak represents a more significant increase in health risks for monophasic curve carriers than for biphasic curve carriers. A triphasic curve with a delayed peak appears to be slightly less beneficial compared to the biphasic one yet significantly healthier compared to a monophasic curve with a delayed peak.

**Table 5:** (**a**) Anthropometric and metabolic characterization of women divided by the shift of the maximum peak of the glycemic curve. (**b**) OGTT descriptions of women divided by the shift of the maximum peak of the glycemic curve. (**c**) Glucose homeostasis of women divided by the shift of the maximum peak of the glycemic curve.

**(a)**									
**Women**	**Monophasic Curve** **n = 533**			**Biphasic Curve** **n = 147**			**Triphasic Curve** **n = 308**		
	**Maximum Peak during the OGTT**			**Maximum Peak during the OGTT**			**Maximum Peak during the OGTT**		
	**at 30 min** **n = 316**	**after 30 min** **n = 217**	***p*-Level**	**at 30 min ** **n = 124**	**after 30 min** **n = 23**	***p*-Level**	**at 30 min ** **n = 249**	**after 30 min** **n = 59**	***p*-Level**
NGT/IFG + IGT/T2DM	286/30/0	148/59/10		118/5/1	20/3/0		237/12/0	49/9/1	
Anthropometric Parameters									
Age [years]	33.5 (26.8, 40.9)	34.6 (29.4, 41.1)	0.103	31.8 (25.2, 36.9)	39.2 (30.3, 41)	0.011	32.6 (27.1, 38.1)	34.6 (30.7, 39.7)	0.021
BMI [kg/m^2^]	23.7 (21, 27.5)	25.6 (21.1, 30.2)	0.007	22.7 (20.3, 27)	24.3 (20, 28.1)	0.819	22.8 (20.7, 25.8)	23.5 (21, 27.3)	0.271
BAI [%]	28.1 (25.4, 32.1)	29.9 (26.1, 34.1)	0.006	26.7 (24.7, 31)	27.9 (25.3, 30)	0.806	27.4 (25.3, 30.6)	28.1 (25.7, 31.9)	0.605
Systolic blood pressure [mmHg]	114 (105, 124)	115 (108, 126)	0.179	111 (102, 120)	120 (107, 127)	0.031	111 (103, 119)	112 (105, 122)	0.661
Diastolic blood pressure [mmHg]	72 (65.5, 79.5)	73 (67, 81)	0.078	70 (64, 77)	74 (65.5, 82)	0.034	70 (64.8, 79)	73 (67, 77)	0.289
Abdominal circumference [cm]	86 (78.3, 95.2)	90.3 (79.9, 99.1)	0.007	83 (77.1, 95.6)	88.1 (76.3, 95.5)	0.787	83.6 (77.6, 90.9)	85.5 (80.9, 93.5)	0.016
Hip circumference [cm]	101 (95, 108)	103 (96, 111)	0.053	99.5 (94.5, 106)	100 (94.8, 103)	0.535	99 (93.9, 105)	101 (95.8, 107)	0.326
Waist circumference [cm]	76 (69.7, 86)	80.8 (71.9, 92.1)	0.002	73 (69.3, 84.5)	75.5 (70.3, 85.5)	0.744	74 (68.8, 80)	75.1 (71, 85.3)	0.049
WHR	0.766 (0.728, 0.812)	0.792 (0.743, 0.836)	0.002	0.756 (0.718, 0.794)	0.756 (0.719, 0.859)	0.143	0.75 (0.721, 0.791)	0.767 (0.727, 0.816)	0.022
Biochemical Parameters									
Total cholesterol [mmol/L]	4.57 (4.08, 5.2)	4.74 (4.09, 5.28)	0.343	4.48 (4.07, 5.03)	4.58 (3.91, 5.25)	0.828	4.52 (3.97, 5.03)	4.66 (4.05, 5.26)	0.167
HDL cholesterol [mmol/L]	1.57 (1.36, 1.79)	1.5 (1.24, 1.8)	0.091	1.59 (1.38, 1.88)	1.5 (1.22, 1.81)	0.217	1.61 (1.37, 1.87)	1.55 (1.29, 1.82)	0.292
LDL cholesterol [mmol/L]	2.6 (2.12, 3.11)	2.63 (2.06, 3.14)	0.873	2.41 (2.07, 2.94)	2.56 (2.16, 2.88)	0.432	2.4 (2.05, 2.94)	2.64 (2.09, 3.3)	0.102
Triacylglycerols [mmol/L]	0.86 (0.64, 1.16)	1.03 (0.66, 1.48)	0.001	0.75 (0.595, 1.11)	0.76 (0.56, 1.2)	0.579	0.785 (0.57, 1.06)	0.705 (0.543, 1.06)	0.696
Urea [mmol/L]	4.1 (3.6, 5)	4.38 (3.7, 5.1)	0.262	4.2 (3.5, 4.8)	4.7 (3.8, 5.08)	0.159	4.2 (3.59, 5.06)	4.37 (4, 5)	0.187
Uric acid [umol/L]	248 (223, 286)	272 (233, 305)	<0.001	255 (207, 290)	274 (237, 305)	0.213	246 (214, 292)	251 (218, 284)	0.945
Creatinine [umol/L]	65 (59, 72.4)	65.9 (58.6, 73)	0.777	64.4 (57.1, 71)	65.5 (62.9, 71.8)	0.169	65 (58, 71)	62 (57, 67)	0.035
TSH [mIU/L]	2.25 (1.52, 3.28)	2.25 (1.68, 3.3)	0.383	2.4 (1.72, 3.27)	2.19 (1.69, 3.29)	0.925	2.41 (1.68, 3.12)	2.19 (1.48, 3.01)	0.038
Free T4 [pmol/L]	15.1 (13.9, 16.5)	15.1 (13.7, 16.3)	0.863	15 (13.6, 16.5)	15 (13.6, 16.2)	0.899	15.1 (13.4, 16.7)	14.6 (13.8, 16)	0.9
Free T3 [pmol/L]	4.81 (4.37, 5.31)	4.89 (4.47, 5.41)	0.267	4.79 (4.49, 5.49)	4.71 (4.31, 5.11)	0.074	4.74 (4.32, 5.24)	4.76 (4.34, 5.03)	0.167
ALT [ukat/L]	0.28 (0.22, 0.36)	0.32 (0.25, 0.44)	<0.001	0.27 (0.23, 0.34)	0.27 (0.21, 0.337)	0.337	0.28 (0.228, 0.35)	0.31 (0.225, 0.365)	0.736
AST [ukat/L]	0.34 (0.29, 0.4)	0.37 (0.3, 0.44)	0.003	0.33 (0.29, 0.4)	0.33 (0.285, 0.358)	0.913	0.33 (0.28, 0.38)	0.34 (0.28, 0.43)	0.786
GGT [ukat/L]	0.21 (0.15, 0.31)	0.25 (0.19, 0.405)	<0.001	0.21 (0.17, 0.28)	0.21 (0.14, 0.3)	0.471	0.2 (0.16, 0.27)	0.21 (0.15, 0.27)	0.84
**(b)**									
**Women**	**Monophasic Curve** **n = 533**			**Biphasic Curve** **n = 147**			**Triphasic Curve** **n = 308**		
	**Maximum peak during the OGTT**			**Maximum peak during the OGTT**			**Maximum peak during the OGTT**		
	**at 30 min** **n = 316**	**after 30 min** **n = 217**	***p*-level**	**at 30 min ** **n = 124**	**after 30 min** **n = 23**	***p*-level**	**at 30 min ** **n = 249**	**after 30 min** **n = 59**	***p*-level**
Ogtt Descriptions									
Glucose 0 min [mmol/L]	4.8 (4.5, 5.1)	4.8 (4.5, 5.4)	0.023	4.75 (4.5, 5)	4.7 (4.35, 5)	0.462	4.7 (4.4, 4.9)	4.9 (4.6, 5.1)	0.012
Glucose 30 min	7.9 (7.1, 8.8)	8.2 (7.1, 9.1)	0.352	7.6 (6.9, 8.53)	8.2 (6.75, 8.5)	0.566	7.2 (6.3, 8)	7.3 (6.25, 8.1)	0.708
Glucose 60 min	7.2 (6.38, 8.2)	9.2 (8.1, 10.6)	<0.001	6.35 (5.38, 7.4)	8.7 (7.85, 9.5)	<0.001	5.8 (5, 6.7)	7.6 (6.45, 8.85)	<0.001
Glucose 90 min	6 (5.3, 7.02)	8.1 (7, 9.5)	<0.001	5.4 (4.4, 5.93)	6.1 (5.9, 7.65)	<0.001	4.9 (4.3, 5.7)	6 (5.25, 7.1)	<0.001
Glucose 120 min	5.45 (4.7, 6.02)	6.7 (5.7, 7.7)	<0.001	4.2 (3.6, 4.93)	5.3 (4.3, 6.3)	<0.001	5.4 (4.8, 6.2)	6.3 (5.8, 7.3)	<0.001
Glucose 150 min	4.3 (3.6, 5)	5.3 (4.4, 6.6)	<0.001	3.3 (2.8, 4)	4.8 (2.8, 5.7)	0.04	4.8 (4.1, 5.4)	5.5 (4.9, 6.2)	<0.001
Glucose 180 min	3.6 (3.2, 4.1)	4 (3.3, 5.1)	<0.001	3.9 (3.6, 4.4)	5 (3.45, 5.75)	0.012	3.9 (3.4, 4.6)	4.4 (4.05, 4.9)	<0.001
C-peptide 0 min [nmol/L]	0.58 (0.48, 0.74)	0.67 (0.52, 0.91)	0.001	0.58 (0.48, 0.74)	0.57 (0.455, 0.775)	0.841	0.58 (0.49, 0.73)	0.56 (0.45, 0.685)	0.388
C-peptide 30 min	2.13 (1.71, 2.66)	1.91 (1.46, 2.55)	<0.001	2.15 (1.82, 2.79)	1.83 (1.43, 2.29)	0.005	2.14 (1.74, 2.56)	1.67 (1.35, 2.01)	<0.001
C-peptide 60 min	2.83 (2.29, 3.49)	2.9 (2.31, 3.71)	0.444	2.8 (2.38, 3.33)	3.14 (2.62, 3.7)	0.103	2.46 (2.09, 3.02)	2.35 (1.94, 2.9)	0.34
C-peptide 90 min	2.71 (2.2, 3.39)	3.11 (2.58, 4.01)	<0.001	2.52 (2.15, 2.99)	2.75 (2.4, 3.65)	0.033	2.23 (1.81, 2.61)	2.32 (1.92, 2.77)	0.197
C-peptide 120 min	2.25 (1.82, 2.9)	2.88 (2.28, 3.74)	<0.001	1.71 (1.4, 2.22)	2.04 (1.73, 2.82)	<0.001	2.11 (1.72, 2.59)	2.25 (1.85, 2.96)	0.112
C-peptide 150 min	1.55 (1.22, 2.08)	2.11 (1.69, 2.85)	<0.001	1.1 (0.8, 1.42)	1.43 (1.02, 2.4)	<0.001	1.65 (1.35, 2.06)	1.81 (1.55, 2.4)	0.019
C-peptide 180 min	0.995 (0.78, 1.32)	1.42 (1.05, 1.99)	<0.001	0.75 (0.587, 1.08)	1.19 (0.715, 1.99)	<0.001	1.04 (0.83, 1.33)	1.21 (0.93, 1.58)	0.006
Insulin 0 min [mIU/L]	6.15 (4.5, 9.22)	7 (4.5, 12.9)	0.004	6.05 (4.4, 8.88)	5.6 (3.75, 8.6)	0.774	5.9 (4.4, 8.4)	5.7 (4.05, 8.2)	0.571
Insulin 30 min	48.7 (34.6, 72.3)	43.7 (28.3, 66.6)	0.006	51.9 (39.5, 71.3)	40.7 (23.2, 57.7)	0.002	52.1 (36.5, 69.5)	37.9 (25.6, 49.6)	<0.001
Insulin 60 min	53 (35.9, 79.6)	60.9 (40.7, 98.7)	0.002	53.9 (39.7, 72.8)	58 (41.4, 76.8)	0.389	44.4 (31.4, 63.3)	48.2 (36.4, 61.9)	0.096
Insulin 90 min	40.5 (28.1, 62.9)	57.9 (39.1, 91.6)	<0.001	37.4 (26.6, 53)	49 (29.6, 72.9)	0.089	32.4 (22.1, 45.4)	37.5 (26, 50.6)	0.06
Insulin 120 min	26.3 (17.4, 41.6)	43 (27.5, 81.6)	<0.001	18.6 (10.4, 26.9)	20.8 (13.3, 40.3)	0.018	28.8 (20.9, 40.9)	33 (22.9, 46.9)	0.05
Insulin 150 min	11.3 (7.28, 21.3)	24.6 (13.2, 45.3)	<0.001	6.55 (3.8, 12.5)	13.5 (4.85, 24.4)	0.002	16.7 (9.8, 25.6)	18.7 (13.4, 35.4)	0.002
Insulin 180 min	5.7 (3.9, 8.63)	9.5 (5.9, 21.1)	<0.001	4.6 (3, 9.13)	10.5 (4.35, 18.1)	0.004	6.1 (3.8, 10.3)	7.4 (4.95, 13.3)	0.002
AUC gluc 30 min	189 (176, 207)	195 (177, 215)	0.087	185 (171, 201)	188 (172, 205)	0.678	179 (162, 192)	180 (164, 197)	0.141
AUC gluc	1050 (958, 1170)	1260 (1140, 1420)	<0.001	939 (847, 1030)	1090 (968, 1240)	<0.001	972 (881, 1080)	1110 (1010, 1220)	<0.001
ΔAUC gluc	201 (116, 297)	378 (275, 513)	<0.001	123 (52.5, 215)	260 (207, 383)	<0.001	141 (79.5, 219)	264 (137, 381)	<0.001
AUC ins 30 min	5050 (3550, 7400)	4670 (3030, 6970)	0.041	5390 (3950, 7240)	4170 (2300, 6110)	0.002	5270 (3800, 7050)	3750 (2850, 4960)	<0.001
AUC ins	34,600 (25,100, 50,400)	44,000 (29,600, 72,400)	<0.001	34,200 (25,900, 42,100)	31,200 (25,700, 47,900)	0.489	32,700 (25,200, 43,800)	33,100 (24,700, 47,900)	0.633
ΔAUC ins	27,500 (19,700, 41,600)	36,800 (24,600, 57,900)	<0.001	27,100 (19,400, 33,200)	25,300 (21,400, 36,000)	0.216	25,700 (19,600, 36,000)	26,700 (19,700, 40,100)	0.388
AUC cp 30 min	41,300 (33,200, 50,600)	38,700 (30,500, 50,900)	0.05	41,900 (35,500, 51,900)	34,500 (29,000, 46,600)	0.02	41,600 (34,500, 48,800)	33,000 (27,900, 39,000)	<0.001
AUC cp	374 × 10^3^ (317, 453) × 10^3^	425 × 10^3^ (338, 546) × 10^3^	<0.001	343 × 10^3^ (291, 393) × 10^3^	344 × 10^3^ (310, 471) × 10^3^	0.043	346 × 10^3^ (295, 410) × 10^3^	333 × 10^3^ (286, 416) × 10^3^	0.593
ΔAUC cp	265 × 10^3^ (215, 325) × 10^3^	300 × 10^3^ (244, 387) × 10^3^	<0.001	233 × 10^3^ (192, 271) × 10^3^	262 × 10^3^ (217, 343) × 10^3^	0.006	236 × 10^3^ (198, 285) × 10^3^	241 × 10^3^ (190, 305) × 10^3^	0.863
**(c)**									
**Women**	**Monophasic curve** **n = 533**			**Biphasic curve** **n = 147**			**Triphasic curve** **n = 308**		
	**Maximum peak during the OGTT**			**Maximum peak during the OGTT**			**Maximum peak during the OGTT**		
	**at 30 min** **n = 316**	**after 30 min** **n = 217**	***p*-level**	**at 30 min ** **n = 124**	**after 30 min** **n = 23**	***p*-level**	**at 30 min ** **n = 249**	**after 30 min** **n = 59**	***p*-level**
Insulin Sensitivity/Resistance									
HOMA-R	1.3 (0.905, 1.98)	1.52 (0.919, 2.9)	0.002	1.28 (0.917, 1.88)	1.1 (0.749, 1.84)	0.698	1.24 (0.841, 1.8)	1.22 (0.808, 1.74)	0.966
QUICKI	0.367 (0.344, 0.39)	0.358 (0.326, 0.389)	0.002	0.368 (0.347, 0.389)	0.377 (0.349, 0.403)	0.663	0.37 (0.349, 0.395)	0.371 (0.351, 0.398)	0.758
OGIS 2h	459 (418, 498)	429 (368, 470)	<0.001	482 (455, 519)	467 (441, 511)	0.3	466 (433, 505)	438 (404, 478)	<0.001
OGIS 3h	522 (473, 562)	471 (407, 526)	<0.001	522 (463, 560)	498 (431, 545)	0.104	508 (472, 556)	489 (443, 513)	<0.001
ISIcomp	8.3 (5.58, 11.3)	6.11 (3.26, 9.75)	<0.001	8.89 (6.72, 11.3)	9.28 (5.65, 11.3)	0.413	8.59 (6.3, 11.8)	9.17 (5.75, 11)	0.242
MCRest	9.86 (8.34, 10.8)	8.39 (6.06, 9.86)	<0.001	10.6 (9.5, 11.3)	10.2 (8.44, 10.7)	0.107	10.3 (9.19, 11)	9.63 (8.49, 10.8)	0.005
Si(oral)	0.152 (0.085, 0.241)	0.081 (0.039, 0.147)	<0.001	0.205 (0.137, 0.303)	0.163 (0.086, 0.224)	0.023	0.183 (0.117, 0.291)	0.141 (0.089, 0.233)	0.001
PREDIM	7.07 (5.53, 8.9)	5.64 (3.79, 7.64)	<0.001	7.87 (6.52, 10)	7.77 (5.73, 9.42)	0.196	7.24 (5.82, 8.84)	6.37 (5.53, 8.09)	0.013
Beta Cell Function									
HOMA-beta	103 (71.3, 158)	105 (72.3, 171)	0.416	100 (73.2, 154)	102 (69.7, 216)	0.659	108 (78.2, 148)	90.9 (64.4, 124)	0.004
Ins0/Gluc0	7.62 (5.74, 11)	8.53 (5.87, 13.9)	0.018	7.54 (5.72, 11.3)	8.04 (5.12, 11.1)	0.899	7.7 (6, 10.4)	6.76 (5.23, 9.84)	0.227
Cp0/Gluc0	420 (317, 607)	470 (324, 767)	0.019	416 (315, 624)	444 (282, 615)	0.898	425 (331, 572)	373 (289, 543)	0.226
IGI	82.6 (54.5, 132)	69.2 (44.4, 117)	0.033	104 (67, 169)	55.9 (37.7, 93)	<0.001	117 (81.2, 166)	80.4 (59.3, 139)	<0.001
IGI simplified	38.3 (26.4, 54.4)	31 (21.7, 51.4)	0.001	42.2 (29.1, 57.7)	29.4 (18.5, 41.4)	0.002	43.2 (33.3, 57.5)	32.1 (21.9, 43)	<0.001
IGI cp	505 (357, 697)	388 (281, 581)	<0.001	584 (416, 902)	368 (307, 591)	0.001	625 (468, 861)	450 (349, 675)	<0.001
IGI simplified cp	2110 (1450, 3000)	1710 (1200, 2840)	0.001	2330 (1600, 3180)	1620 (1020, 2290)	0.002	2380 (1840, 3170)	1770 (1210, 2370)	<0.001
AUC ins/AUC gluc	33.2 (24.5, 48.3)	34.1 (25.3, 53.3)	0.102	35.9 (27.5, 44.5)	32 (24.4, 45.7)	0.507	34.3 (27.5, 44.7)	30.8 (23, 38.3)	0.027
ΔAUC ins/ΔAUC gluc	158 (92.1, 291)	106 (66.5, 176)	<0.001	227 (143, 555)	121 (68.6, 148)	<0.001	209 (127, 318)	118 (86, 202)	<0.001
AUC cp/AUC gluc	362 (292, 431)	336 (273, 435)	0.05	354 (306, 440)	336 (291, 456)	0.635	367 (300, 411)	310 (256, 363)	<0.001
ΔAUC cp/ΔAUC gluc	1410 (888, 2190)	841 (602, 1150)	<0.001	1840 (1280, 4220)	1070 (657, 1360)	<0.001	1700 (1120, 2890)	1030 (727, 1770)	<0.001
Disposition Indices									
IGI × ISIcomp	265 (213, 328)	214 (171, 271)	<0.001	300 (247, 383)	287 (199, 346)	0.057	296 (248, 358)	246 (201, 304)	<0.001
OGIS 3h × AUCins	17.9 × 10^6^ (13.6, 25.6) × 10^6^	20.7 × 10^6^ (15.3, 28.6) × 10^6^	<0.001	17.4 × 10^6^ (13.6, 20.9) × 10^6^	16.4 × 10^6^ (12.7, 22.9) × 10^7^	0.705	16.8 × 10^7^ (13.6, 22.7) × 10^6^	16.0 × 10^7^ (12.8, 21.0) × 10^7^	0.613
Adaptation Index									
OGIS 3h × AUCcp	19.2 × 10^7^ (16.5, 22.9) × 10^7^	19.0 × 10^7^ (16.8, 23.3) × 10^7^	0.424	17.3 × 10^7^ (14.4, 20.6) × 10^7^	19.3 × 10^7^ (16.1, 22.3) × 10^7^	0.254	17.7 × 10^7^ (15.2, 20.5) × 10^7^	15.9 × 10^7^ (14.4, 19.2) × 10^7^	0.025
Hepatic Extraction									
HE	68.3 (61.4, 74.4)	64.9 (54.7, 71.5)	<0.001	67.2 (61.7, 73.4)	71.7 (63.4, 74.5)	0.548	68.1 (62.4, 72.6)	67.2 (60.2, 72.8)	0.535

Data are given as median (95% LCL; 95% UCL) and *p*-levels according to ANOVA test. Explanatory notes and abbreviations for the tabulated parameters are available in Appendix A, Table A1.

**Table 6:** (**a**) Anthropometric and metabolic characterization of men divided by the shift of the maximum peak of the glycemic curve. (**b**) OGTT descriptions of men divided by the shift of the maximum peak of the glycemic curve. (**c**) Glucose homeostasis of men divided by the shift of the maximum peak of the glycemic curve.

**(a)**									
**Men**	**Monophasic Curve** **n = 100**			**Biphasic Curve** **n = 74**			**Triphasic Curve** **n = 43**		
	**Maximum Peak during the OGTT**			**Maximum Peak during the OGTT**			**Maximum Peak during the OGTT**		
	**at 30 min** **n = 53**	**after 30 min** **n = 47**	***p*-Level**	**at 30 min ** **n = 60**	**after 30 min** **n = 14**	***p*-Level**	**at 30 min ** **n = 31**	**after 30 min** **n = 12**	***p*-Level**
NGT/IFG + IGT/T2DM	41/10/2	32/12/3		59/1/0	13/1/0		26/ 5/0	10/2/0	
Anthropometric Parameters									
Age [years]	35.6 (27, 44.5)	40.3 (25.8, 52.2)	0.373	26.8 (23.7, 36.8)	31.4 (24, 40.4)	0.388	38.4 (30.8, 45.9)	34.6 (31.5, 49.5)	0.995
BMI [kg/m2]	24.8 (23, 28.1)	26.5 (23.5, 31.6)	0.149	23.4 (21.7, 26.3)	24.6 (23.8, 27.8)	0.149	25.3 (23.7, 27.6)	24.6 (22, 26.1)	0.118
BAI [%]	23.7 (22.1, 25.5)	24.4 (21.8, 27.2)	0.556	22.3 (20.3, 23.4)	22.7 (21.6, 25.2)	0.07	23.4 (21.3, 25.2)	23 (20.2, 23.9)	0.21
Systolic blood pressure [mmHg]	128 (115, 139)	129 (117, 140)	0.963	120 (114, 129)	115 (110, 120)	0.067	121 (112, 133)	122 (116, 126)	0.925
Diastolic blood pressure [mmHg]	77 (70, 87)	77 (70, 85.5)	0.775	73 (69.5, 78.5)	69 (67, 75)	0.483	75 (67, 81)	66.5 (63.3, 78.3)	0.348
Abdominal circumference [cm]	89.3 (85.4, 100)	96 (84.7, 108)	0.201	85.4 (79.2, 92)	88.6 (85.5, 95.3)	0.138	92.1 (86.7, 97.1)	89.2 (82.5, 95.1)	0.22
Hip circumference [cm]	99.9 (96.6, 104)	102 (95, 109)	0.35	97.3 (94.3, 102)	100 (98.2, 102)	0.228	100 (96.6, 107)	98.3 (90.4, 101)	0.105
Waist circumference [cm]	86.9 (80.2, 95.1)	93.5 (82.1, 106)	0.153	81 (76.4, 88.4)	84.2 (80, 91.6)	0.186	88.2 (83.1, 93.6)	87.1 (80.3, 93.4)	0.443
WHR	0.865 (0.821, 0.919)	0.901 (0.852, 0.979)	0.072	0.836 (0.802, 0.883)	0.848 (0.814, 0.909)	0.309	0.876 (0.839, 0.926)	0.888 (0.844, 0.92)	0.47
Biochemical parameters									
Total cholesterol [mmol/L]	4.95 (4.34, 5.41)	4.75 (4.17, 5.43)	0.574	4.54 (3.82, 5.03)	4.3 (3.51, 4.56)	0.459	4.84 (4.47, 5.32)	4.98 (3.87, 5.65)	0.777
HDL cholesterol [mmol/L]	1.3 (1.11, 1.47)	1.21 (1.02, 1.51)	0.503	1.26 (1.07, 1.49)	1.26 (0.943, 1.39)	0.294	1.24 (1.11, 1.54)	1.15 (1.01, 1.51)	0.491
LDL cholesterol [mmol/L]	3 (2.43, 3.4)	2.8 (2.18, 3.36)	0.25	2.62 (2.04, 3.17)	2.46 (2.2, 2.83)	0.421	2.99 (2.4, 3.34)	2.79 (2.28, 3.5)	0.888
Triacylglycerols [mmol/L]	1 (0.74, 1.65)	1.16 (0.825, 2.15)	0.103	1 (0.672, 1.44)	1.05 (0.845, 1.31)	0.365	0.94 (0.765, 1.63)	1.18 (0.89, 1.47)	0.415
Urea [mmol/L]	4.8 (4, 5.7)	5 (4.6, 5.97)	0.1	4.9 (4.12, 6.1)	4.85 (4.4, 5.15)	0.888	5.3 (4.6, 5.95)	4.69 (4.03, 5.33)	0.079
Uric acid [umol/L]	345 (297, 404)	337 (289, 397)	0.466	329 (296, 361)	324 (298, 363)	0.394	305 (291, 353)	331 (261, 358)	0.829
Creatinine [umol/L]	79.6 (72.8, 89)	80.5 (75.8, 89.5)	0.414	82.2 (74.8, 89.4)	80.4 (69.6, 84.8)	0.205	79 (74, 92)	74.9 (70.2, 82.5)	0.25
TSH [mIU/L]	2.16 (1.38, 2.73)	2.24 (1.52, 2.75)	0.854	2.01 (1.52, 3.01)	2.22 (1.93, 2.77)	0.492	1.82 (1.39, 2.31)	1.92 (1.34, 2.88)	0.72
Free T4 [pmol/L]	15.9 (14.7, 17)	15.4 (13.9, 17.7)	0.942	15.8 (14.4, 17.6)	15.9 (14.9, 16.8)	0.636	15.5 (14.3, 17.4)	17.1 (15.5, 19.6)	0.039
Free T3 [pmol/L]	5.17 (4.77, 5.66)	5.13 (4.6, 5.67)	0.551	5.38 (4.96, 6.01)	5.59 (5.07, 5.88)	0.767	5.19 (4.91, 5.57)	5.27 (5.03, 5.47)	0.536
ALT [ukat/L]	0.45 (0.35, 0.55)	0.43 (0.32, 0.54)	0.930	0.39 (0.308, 0.623)	0.485 (0.36, 0.538)	0.546	0.39 (0.315, 0.51)	0.325 (0.21, 0.512)	0.175
AST [ukat/L]	0.41 (0.37, 0.47)	0.4 (0.355, 0.52)	0.826	0.44 (0.373, 0.517)	0.38 (0.325, 0.51)	0.139	0.41 (0.36, 0.49)	0.36 (0.32, 0.585)	0.511
GGT [ukat/L]	0.36 (0.28, 0.53)	0.465 (0.292, 0.6)	0.202	0.31 (0.21, 0.425)	0.375 (0.263, 0.53)	0.093	0.38 (0.245, 0.555)	0.24 (0.215, 0.648)	0.623
**(b)**									
**Men**	**Monophasic curve** **n = 100**			**Biphasic curve** **n = 74**			**Triphasic curve** **n = 43**		
	**Maximum peak during the OGTT**			**Maximum peak during the OGTT**			**Maximum peak during the OGTT**		
	**at 30 min** **n = 53**	**after 30 min** **n = 47**	***p*-level**	**at 30 min ** **n = 60**	**after 30 min** **n = 14**	***p*-level**	**at 30 min ** **n = 31**	**after 30 min** **n = 12**	***p*-level**
Ogtt descriptions									
Glucose 0 min [mmol/L]	5 (4.7, 5.5)	5 (4.7, 5.4)	0.976	4.7 (4.4, 4.93)	4.85 (4.6, 5.28)	0.11	5 (4.8, 5.3)	4.7 (4.5, 5.08)	0.565
Glucose 30 min	8.5 (7.5, 9.1)	8.5 (7.5, 9.85)	0.444	7.75 (6.8, 8.7)	7.95 (7.48, 8.68)	0.476	7.8 (7.05, 8.45)	7.65 (6.65, 8.5)	0.987
Glucose 60 min	7.6 (6.4, 9.1)	9.7 (8.25, 11)	<0.001	6 (5.2, 7.4)	8.45 (8.03, 9.45)	<0.001	6.5 (5.85, 7.45)	7.85 (6.42, 9.42)	0.03
Glucose 90 min	6.4 (5.9, 7.4)	7.8 (7.15, 9.9)	<0.001	4.65 (4.1, 5.4)	6.25 (5.25, 7)	<0.001	5.1 (4.65, 6)	5.8 (5.05, 6.43)	0.205
Glucose 120 min	5.4 (4.8, 6.1)	6.5 (5.3, 7.5)	<0.001	3.7 (3.3, 4.12)	4.5 (3.93, 4.85)	0.001	5.5 (4.85, 6.4)	5.75 (4.28, 6.1)	0.734
Glucose 150 min	4.4 (3.8, 5)	4.6 (4.1, 5.4)	0.034	3.3 (2.98, 3.52)	3.4 (3.02, 3.57)	0.533	4.6 (4.2, 5.15)	4.25 (3.63, 4.9)	0.279
Glucose 180 min	3.9 (3.6, 4.5)	3.8 (3.4, 4.6)	0.941	4 (3.6, 4.2)	4.1 (3.6, 4.42)	0.161	4.1 (3.6, 4.6)	3.65 (3.15, 4.03)	0.212
C-peptide 0 min [nmol/L]	0.61 (0.48, 0.86)	0.71 (0.485, 1.06)	0.188	0.515 (0.4, 0.632)	0.555 (0.465, 0.777)	0.115	0.57 (0.445, 0.75)	0.565 (0.492, 0.638)	0.752
C-peptide 30 min	2.17 (1.85, 2.75)	2.1 (1.68, 2.73)	0.137	2.43 (1.98, 2.8)	2.28 (1.7, 3.29)	0.946	2.05 (1.42, 2.62)	1.7 (1.52, 1.91)	0.391
C-peptide 60 min	3.01 (2.51, 3.94)	3.29 (2.44, 3.71)	0.97	2.71 (2.23, 3.52)	3.44 (2.75, 3.99)	0.054	2.13 (1.55, 3.1)	2.62 (2.16, 2.76)	0.425
C-peptide 90 min	2.8 (2.12, 3.63)	3.26 (2.68, 4.18)	0.028	2.23 (1.78, 2.84)	3.38 (2.75, 3.85)	0.001	1.93 (1.5, 2.7)	2.2 (1.84, 2.5)	0.427
C-peptide 120 min	2.19 (1.51, 2.88)	2.83 (2.08, 3.5)	0.006	1.47 (0.978, 1.76)	2.12 (1.58, 2.75)	0.001	1.68 (1.26, 2.56)	1.86 (1.41, 2.46)	0.604
C-peptide 150 min	1.3 (1.03, 1.86)	1.85 (1.34, 2.77)	<0.001	0.78 (0.6, 0.955)	1.19 (0.835, 1.58)	0.001	1.25 (0.835, 1.78)	1.35 (0.793, 1.66)	0.94
C-peptide 180 min	0.87 (0.64, 1.39)	1.15 (0.91, 1.73)	0.001	0.55 (0.465, 0.692)	0.77 (0.62, 1.22)	<0.001	0.79 (0.59, 1.11)	0.835 (0.61, 1.09)	0.855
Insulin 0 min [mIU/L]	6.7 (5.3, 10.5)	7.1 (4.65, 11)	0.945	5.55 (3.9, 7.43)	6.25 (4.43, 12.1)	0.078	6.2 (4.6, 9.35)	4.7 (3.2, 6.47)	0.178
Insulin 30 min	52.9 (37, 69.6)	42.4 (28.4, 61)	0.197	47.1 (37.6, 63.3)	40 (32.1, 61.2)	0.846	40.7 (27.1, 72.9)	30.5 (24.6, 61.3)	0.412
Insulin 60 min	55.2 (40.5, 81.9)	60.2 (41.1, 98.4)	0.417	38.3 (27.8, 63.7)	53.9 (46.3, 65.1)	0.119	35.7 (19.7, 60.6)	45 (33.8, 62.4)	0.393
Insulin 90 min	38.8 (25.2, 59)	53.6 (35.8, 92.2)	0.008	23.6 (17.2, 39.5)	43 (24.5, 62.3)	0.028	25 (15.6, 43)	26.6 (19.8, 42.8)	0.698
Insulin 120 min	21.3 (11.4, 38.1)	35.4 (18.8, 69.3)	0.003	11.4 (6.17, 14.2)	18.5 (12.5, 29.6)	0.002	17.9 (10.5, 36.6)	23.8 (10.4, 40.2)	0.948
Insulin 150 min	7.9 (4.4, 18.9)	14.7 (9.85, 37.9)	0.001	3.95 (2.9, 6.85)	5.95 (4.43, 11.2)	0.021	9.4 (5.1, 21.6)	7.8 (4.98, 17.5)	0.931
Insulin 180 min	4.7 (3.1, 8.9)	8 (3.9, 16.2)	0.011	3.24 (2.48, 4.85)	4.45 (3.15, 9.95)	0.097	4.1 (2.35, 6.6)	4.2 (3.15, 4.62)	0.973
AUC gluc 30 min	203 (185, 224)	203 (185, 223)	0.746	182 (171, 206)	193 (183, 204)	0.285	192 (179, 206)	186 (174, 198)	0.139
AUC gluc	1110 (1000, 1210)	1230 (1130, 1430)	<0.001	896 (820, 974)	1050 (1000, 1130)	<0.001	1030 (943, 1090)	1020 (957, 1200)	0.642
ΔAUC gluc	197 (128, 281)	356 (257, 459)	<0.001	101 (45, 171)	176 (102, 259)	0.012	155 (76.5, 236)	213 (137, 311)	0.066
AUC ins 30 min	5230 (3930, 7070)	4500 (3020, 6560)	0.22	4670 (3740, 6370)	4550 (3380, 6540)	0.709	4240 (2830, 7300)	3150 (2590, 5990)	0.351
AUC ins	32,000 (24,000, 51,700)	42,400 (26,500, 64,300)	0.143	25,300 (18,500, 32,400)	27,800 (25,200, 46,600)	0.07	29,300 (17,700, 43,200)	29,900 (19,400, 35,000)	0.905
ΔAUC ins	26,400 (17,900, 40,300)	30,000 (20,400, 52,200)	0.054	18,800 (13,100, 26,500)	22,900 (20,200, 30,300)	0.079	19,000 (12,000, 33,700)	22,000 (15,600, 30,000)	0.731
AUC cp 30 min	43,200 (35,600, 57,500)	41,000 (33,600, 55,700)	0.494	45,200 (36,300, 51,900)	42,200 (33,200, 63,300)	0.688	41,100 (28,100, 51,500)	35,400 (30,900, 38,300)	0.415
AUC cp	379 × 10^3^ (291, 483) × 10^3^	440 × 10^3^ (331, 535) × 10^3^	0.067	308 × 10^3^ (254, 379) × 10^3^	441 × 10^3^ (308, 457) × 10^3^	0.008	294 × 10^3^ (234, 406) × 10^3^	312 × 10^3^ (246, 380) × 10^3^	0.789
ΔAUC cp	260 × 10^3^ (202, 321) × 10^3^	284 × 10^3^ (232, 362) × 10^3^	0.200	202 × 10^3^ (167, 261) × 10^3^	295 × 10^3^ (216, 355) × 10^3^	<0.001	203 × 10^3^ (144, 257) × 10^3^	202 × 10^3^ (172, 268) × 10^3^	0.641
**(c)**									
**Men**	**Monophasic curve** **n = 100**			**Biphasic curve** **n = 74**			**Triphasic curve** **n = 43**		
	**Maximum peak during the OGTT**			**Maximum peak during the OGTT**			**Maximum peak during the OGTT**		
	**at 30 min** **n = 53**	**after 30 min** **n = 47**	***p*-level**	**at 30 min ** **n = 60**	**after 30 min** **n = 14**	***p*-level**	**at 30 min ** **n = 31**	**after 30 min** **n = 12**	***p*-level**
Insulin Sensitivity/Resistance									
HOMA-R	1.51 (1.11, 2.7)	1.53 (0.977, 2.61)	0.893	1.15 (0.761, 1.54)	1.38 (0.91, 2.38)	0.048	1.42 (1.01, 2)	1.09 (0.683, 1.4)	0.113
QUICKI	0.359 (0.329, 0.377)	0.358 (0.331, 0.385)	0.948	0.375 (0.358, 0.402)	0.365 (0.336, 0.39)	0.053	0.362 (0.344, 0.383)	0.379 (0.364, 0.41)	0.129
OGIS 2h	441 (393, 483)	425 (377, 468)	0.14	500 (465, 527)	482 (429, 494)	0.016	440 (406, 468)	448 (437, 489)	0.101
OGIS 3h	482 (426, 529)	465 (392, 529)	0.445	500 (476, 532)	494 (424, 508)	0.056	471 (441, 512)	496 (449, 541)	0.086
ISIcomp	8.21 (4.57, 10.8)	5.59 (4.09, 9.83)	0.425	10.7 (8.49, 15.1)	9.23 (5.05, 12)	0.014	9.18 (6.23, 12.7)	9.94 (7.88, 15.9)	0.518
MCRest	9.72 (8.19, 10.5)	8.34 (5.58, 9.44)	0.005	10.8 (10, 11.3)	10.2 (8.95, 10.5)	0.001	9.7 (8.87, 10.6)	10.1 (9.12, 10.5)	0.404
Si(oral)	0.117 (0.0616, 0.186)	0.076 (0.026, 0.123)	0.003	0.24 (0.146, 0.4)	0.145 (0.11, 0.186)	0.02	0.194 (0.101, 0.329)	0.175 (0.092, 0.239)	0.742
PREDIM	6.66 (4.72, 7.64)	5.57 (3.79, 7.39)	0.3	8.2 (7.14, 10.3)	7.22 (5.81, 9.34)	0.075	6.53 (5.49, 7.44)	7.24 (6.53, 8.31)	0.14
Beta Cell Function									
HOMA-beta	105 (72.5, 143)	106 (66.4, 133)	0.868	104 (63.1, 141)	84 (58.7, 178)	0.542	88.2 (58.1, 133)	79 (55.5, 116)	0.843
Ins0/Gluc0	8.09 (6.52, 13.3)	8.63 (5.75, 13.2)	0.945	6.91 (4.99, 9.77)	7.99 (5.05, 15.6)	0.11	7.58 (5.53, 11.6)	6.27 (4.46, 7.96)	0.232
Cp0/Gluc0	446 (360, 734)	476 (317, 727)	0.944	381 (276, 539)	441 (279, 863)	0.11	418 (305, 638)	346 (246, 439)	0.232
IGI	80.4 (62.7, 108)	59.9 (43.8, 112)	0.14	84.5 (63.8, 122)	73.6 (44.4, 186)	0.676	83.3 (53.5, 157)	61 (47.4, 103)	0.033
IGI simplified	35.7 (28.5, 52.7)	28.7 (20.2, 47.5)	0.147	37.3 (27.5, 48.8)	33.6 (23.6, 59.3)	0.924	35.8 (21.6, 58.3)	24.2 (21.7, 45)	0.392
IGI cp	560 (391, 693)	410 (295, 552)	0.001	603 (442, 899)	509 (326, 983)	0.397	496 (335, 713)	401 (325, 559)	0.289
IGI simplified cp	1970 (1570, 2910)	1580 (1110, 2620)	0.147	2060 (1510, 2690)	1850 (1300, 3270)	0.924	1970 (1190, 3210)	1340 (1200, 2480)	0.392
AUC ins/AUC gluc	32 (21.3, 41.6)	32 (21.7, 49.5)	0.699	27.3 (22, 35.6)	27.4 (22.9, 43.1)	0.435	27.6 (16, 40.7)	27.9 (20.9, 31.1)	0.933
ΔAUC ins/ΔAUC gluc	144 (95.6, 228)	107 (67.3, 159)	0.005	220 (138, 457)	131 (88.2, 287)	0.056	172 (99.1, 286)	109 (79.5, 150)	0.181
AUC cp/AUC gluc	339 (287, 417)	343 (275, 422)	0.759	344 (290, 404)	378 (282, 480)	0.298	282 (234, 368)	303 (259, 327)	0.958
ΔAUC cp/ΔAUC gluc	1320 (951, 2200)	886 (634, 1070)	<0.001	2490 (1560, 4780)	1620 (944, 3210)	0.072	1600 (987, 2210)	988 (803, 1350)	0.135
Disposition Indices									
IGI × ISIcomp	214 (182, 263)	211 (146, 257)	0.177	313 (271, 374)	259 (200, 286)	0.002	238 (198, 279)	263 (238, 333)	0.154
OGIS 3h × AUCins	17.1 × 10^6^ (11.4, 21.5) × 10^6^	19.6 × 10^6^ (12.7, 26.9) × 10^6^	0.134	13.0 × 10^6^ (93.2, 15.9) × 10^6^	14.7 × 10^6^ (1.3, 16.0) × 10^6^	0.457	13.8 × 10^6^ (8.2, 19.6) × 10^6^	14.1 × 10^6^ (11.4, 17.8) × 10^6^	0.682
Adaptation Index									
OGIS 3h × AUCcp	17.9 × 10^7^ (15.0, 21.4) × 10^7^	20.5 × 10^7^ (16.3, 23.3) × 10^7^	0.125	15.3 × 10^7^ (12.9, 18.7) × 10^7^	17.5 × 10^7^ (14.8, 22.2) × 10^7^	0.158	13.9 × 10^7^ (12.5, 16.4) × 10^7^	16.5 × 10^7^ (12.7, 19.0) × 10^7^	0.301
Hepatic Extraction									
HE	68.7 (62, 75.4)	67.3 (60, 76)	0.361	72.1 (68.1, 77.5)	69 (66.1, 79.4)	0.721	70.2 (62.8, 77.5)	69.5 (65.8, 75.7)	0.691

Data are given as median (95% LCL; 95% UCL) and *p*-levels according to ANOVA test. Explanatory notes and abbreviations for the tabulated parameters are available in Appendix A, Table A1.

## 4. Discussion

Studies evaluating the shape of glycemic curves are not rare in the literature [20,21,22,36,37], but few deal with 3 h glucose curves. If we focus on studies working with 3 h OGTT [23,26], rare are those in which both women and men are represented. Moreover, most studies evaluating glucose metabolism in both sexes combine female and male cohorts and do not take the specifics into account. In this respect, our research is innovative. Differences between genders and even ethnicity were addressed in a recent study which, however, evaluated the course of OGTT at 2 h only, and glucose metabolism was evaluated only in diabetic patients treated with metformin [38]. Our study provides unique data evaluating both women and men with a wide range of insulin sensitivity, spanning from completely healthy subjects to diabetic patients, and it focuses on revealing metabolic differences between the two genders. The substantial metabolic insight that we found to be different in terms of glucose metabolism was related to insulin secretion, which was significantly lower in men. This confirmed to us that pooling female and male cohorts could introduce a considerable degree of bias into the overall conclusions, and hence, it is appropriate to assess the two sexes separately. On the other side, this approach posed problems, particularly the under-representation of men for certain types of analyses, such as the assessment of health risks associated with the multiphasic course of the glycemic curves. We have, therefore, refrained from drawing conclusions in such situations and offered tabulated trends instead.

The main findings of studies dealing with the shape of glucose trajectory are in agreement with our observations that the monophasic course is associated with lower insulin sensitivity and reduced indices of pancreatic beta cell function [20,21]. However, the total absolute secretion of insulin and C-peptide during the OGTT, as assessed in our study using AUC ins and AUC cp, was the largest in the monophasic curves, both in women and men. This is a consequence of the body’s effort to compensate for the high increase in glucose concentration, which was clearly highest in the monophasic group, as assessed both by absolute glucose levels in the first phase of the test and AUC gluc. In addition, individuals with a monophasic curve, regardless of gender, showed higher concentrations of lipids, higher blood pressure, and larger waist circumference, i.e., parameters falling within the criteria of metabolic syndrome. Compared to monophasic curves, triphasic curves seem to be healthier in terms of biochemical and anthropometric terrain; however, biphasic curves are superior to triphasic ones in this respect. Similar findings were reported in Japanese subjects by Kanauchi et al. [39]. In this study, a greater proportion of monophasic curves was observed in the IGT group, whereas the prevalence of biphasic and triphasic trajectories was much higher in the normotolerant group. An elegant contribution to these conclusions represents the follow-up study conducted by Manco et al. [37]. The shape of the OGTT glucose curve was studied in the EGIR-RISC cohort at baseline and 3 years apart. The baseline monophasic shape was associated with a significant increase in IFG risk, a baseline biphasic shape with a reduced risk of IGT, and a triphasic shape with a reduced risk of IFG after 3 years. Increased risk of IFG was found in people who kept stable monophasic trajectory over time and in switchers from biphasic to monophasic shape glycemic curves. In our cohort, multiphasic curves had the lowest representation, which was only slightly above 4%. The youngest participants in the study, who also showed the best beta cell function, had this shape of curve. As shown in Figure 2, their glucose concentration returned to basal levels the fastest of all the curve categories and fell below this basal level shortly after 1 h of testing. In this respect, our observation is entirely consistent with the findings of the Botnia study [36] conducted on 2445 non-diabetic subjects, which describes that those who returned their plasma glucose concentration below FPG within 60 min had increased IS, greater insulin secretion, and lower risk for future T2DM compared to those whose post-load plasma glucose concentration required 120 min or a longer time to return.

A fundamental finding regarding the glycemic trajectories that has not yet been elaborated in the literature, except for isolated results [40], is the observation of a significantly higher percentage of men showing a biphasic curve, while a higher percentage of women show a triphasic curve. This clearly demonstrates the importance of considering gender when evaluating the shapes of glucose trajectories and other related metabolic parameters. If similar findings are confirmed in further studies on a large set of subjects, it may be appropriate to reconsider the hitherto uniform criteria for men and women with regard to the assessment of glucose tolerance disorders based on the OGTT. The possible explanation for the higher prevalence of men in the group of biphasic curves is a different body composition with a naturally higher proportion of muscle mass in men and a higher proportion of fat mass in women (see BAI in Table 1a). Higher muscle content at the expense of fat correlates positively with muscle insulin sensitivity [41], which, in our study, is the highest in individuals with a biphasic glycemic curve, assessed indirectly through indices of insulin sensitivity.

We consider the lower proportion of men compared to women to be a significant limitation when considering the differences described above. The cause of this disadvantage lies not only in the lower willingness of men to participate in the research project but is also due to the fact that a large proportion of women entering the study were highly motivated. Given the positive history of GDM, a significant part of women welcomed the opportunity to check whether their glucose regulation was in good condition. In fact, those women were advised of their higher risk in a well-developed system of pregnancy counseling centers operating in the Czech Republic. Similarly, women suffering from PCOS who face the same risk were personally motivated regarding glucose metabolism testing. This motivation was absent in men.

The issue of the glycemic peak position within the OGTT curve has already been analyzed in the literature [22,42,43,44,45]. The conclusions of such studies agree with our observation illustrated by Figure 3a,b indicating that the delay of the peak is associated with a higher glucose concentration at this peak and lower insulin sensitivity both in women and men, as demonstrated in Table 5a–c and Table 6a–c. This is especially true in cases of monophasic curves characterized by higher absolute levels of this peak. It is a clinically important finding that could be useful in routine practice, representing a simple and convenient improvement in the diagnostics of glucose tolerance disorders. Today, a 2 h OGTT is commonly used, and impaired glucose tolerance is determined by glycemia at hour 2 of the test. Neither the cost nor the burden on the patient would be significantly increased if glycemic levels were also assessed at the 30th and 60th min of the test, while the predictive value of such a test would be substantially increased. Our approach to the issue is innovative in that we compared how the peak delay varies between the curve categories (i.e., mono-, bi-, tri-, and multiphasic) in terms of the significance of the deterioration in biochemical parameters. We have shown that the delayed peak is associated with an unfavorable biochemical profile mostly in monophasic curves, which, in women, includes, in addition to impaired glycoregulation and other components of metabolic syndrome, parameters indicative of liver function, such as liver enzymes and the rate of hepatic insulin extraction. These findings are in concordance with the published data describing an association of higher serum ALT with prediabetes that was significant only in women [46]. Liver enzymes, especially ALT, appear to be good markers of hepatic fat accumulation and reduced hepatic insulin sensitivity, a condition that precedes the development of T2DM [47]. However, according to the conclusions of one meta-analysis [48], the relationship between ALT and the risk of T2DM may be overestimated. Concerning the health disadvantages of the delayed peak for the other curve categories, monophasic curves were followed by triphasic curves, whereas only mild biochemical deterioration was associated with peak delay in biphasic curves. In multiphasic curves, perhaps due to the lack of sufficient prevalence, we did not observe any adverse health aspects associated with peak delay. Moreover, a delayed peak was very rare in this category. To our best knowledge, the only study combining time to the glucose peak with the shape of the glycemic curve was conducted by La Grasta Sabolić et al. [45] two years ago in obese adolescents with normal glucose tolerance. The conclusion of this study supports our observation by concluding that a combination of the late timing of the glucose peak with the monophasic shape of the curve may indicate early beta cell dysfunction. We fully agree with the message that the combination of a monophasic course and delayed peak poses a health risk requiring intensive lifestyle education and preventative care.

## 5. Conclusions

In conclusion, we have comprehensively assessed the impact of the biochemical background on the dynamics of glucose, insulin, and C-peptide curves during a prolonged OGTT. Our study demonstrates that the shape of the glycemic curve is a reflection of distinct metabolic profiles and varying health conditions. A comparison of glucose processing between women and men led to the original finding. It became clear that the dynamics of glycoregulation differ significantly between the genders. Men show lower total insulin secretion and have differently shaped glycemic curves linked to a more favorable metabolic profile. The novelty of our approach with possible clinical implications lies in the comparison of metabolic backgrounds associated with delayed glycemic peaks in combination with four different curve categories. People with impaired glucose regulation, unfavorable lipid profiles, and other components of metabolic syndrome much more often have a monophasic course of the glycemic curve, with the risk of possible health complications increasing with a delayed peak. Biphasic, triphasic, and multiphasic curves appear to be more convenient in terms of health benefits. Possible delay in the peak, which is much rarer in bi-, tri-, and multiphasic curves than monophasic ones, is not associated with systematic metabolic deterioration and the resulting health risks.

The presented study has a cross-sectional character. However, continuous and repeated examinations of our volunteers in parallel with minimal changes to the investigation protocol over 20 years will also allow us to monitor the evolution of their health status over time. Our future work will, therefore, be focused on the evaluation of such changes; we will trace possible shifts of participants between the four categories of glycemic curves. We also plan to monitor shifts in their glycemic peak, and we intend to analyze the correlation of these changes with the dynamics of the biochemical profile. Such a long-term comparison will provide valuable information on the potential of the glycemic trajectory to predict impaired glucose tolerance and other related metabolic complications in the general population.

## Figures and Tables

**Table 1:** (**a**) Anthropometric and metabolic characterization of the subjects divided by gender. (**b**) Oral glucose tolerance test (OGTT) descriptions of the subjects divided by gender. (**c**) Glucose homeostasis of the subjects divided by gender.

**(a)**			
	**Women** **n = 1035**	**Men** **n = 227**	***p*** **-Level**
NGT/IFG + IGT/T2DM	904/119/12	191/31/5	
Anthropometric Parameters			
Age [years]	33.3 (27.4, 39.4)	34 (25.8, 45.5)	0.298
BMI [kg/m^2^]	23.5 (20.8, 27.7)	24.8 (22.7, 27.5)	<0.001
BAI [%]	28 (25.4, 31.7)	23.2 (21.1, 25.2)	<0.001
Systolic blood pressure [mmHg]	113 (104, 123)	124 (114, 133)	<0.001
Diastolic blood pressure [mmHg]	71 (65, 79)	75 (68, 83)	<0.001
Abdominal circumference [cm]	85 (78.5, 95.2)	89.3 (82.6, 98.9)	<0.001
Hip circumference [cm]	100 (94.5, 107)	99.9 (95.2, 105)	0.571
Waist circumference [cm]	75.5 (69.9, 85.1)	86.3 (79.3, 95.1)	<0.001
WHR	0.764 (0.724, 0.808)	0.864 (0.821, 0.919)	<0.001
Biochemical Parameters			
Total cholesterol [mmol/L]	4.58 (4.03, 5.18)	4.7 (4, 5.23)	0.613
HDL cholesterol [mmol/L]	1.57 (1.33, 1.83)	1.26 (1.04, 1.51)	<0.001
LDL cholesterol [mmol/L]	2.53 (2.07, 3.05)	2.79 (2.2, 3.3)	0.001
Triacylglycerols [mmol/L]	0.83 (0.6, 1.18)	1.01 (0.74, 1.59)	<0.001
Urea [mmol/L]	4.2 (3.6, 5)	5 (4.3, 5.8)	<0.001
Uric acid [umol/L]	254 (220, 292)	330 (293, 384)	<0.001
Creatinine [umol/L]	65 (58.9, 72)	80.5 (73.1, 89)	<0.001
TSH [mIU/L]	2.3 (1.6, 3.2)	2.06 (1.51, 2.82)	0.001
Free T4 [pmol/L]	15 (13.6, 16.5)	15.9 (14.4, 17.4)	<0.001
Free T3 [pmol/L]	4.81 (4.39, 5.32)	5.24 (4.83, 5.74)	<0.001
ALT [ukat/L]	0.29 (0.23, 0.37)	0.42 (0.32, 0.56)	<0.001
AST [ukat/L]	0.34 (0.29, 0.41)	0.41 (0.36, 0.5)	<0.001
GGT [ukat/L]	0.21 (0.16, 0.3)	0.36 (0.24, 0.53)	<0.001
**(b)**			
	**Women** **n = 1035**	**Men** **n = 227**	***p*** **-Level**
Ogtt Descriptions			
Glucose 0 min [mmol/L]	4.7 (4.5, 5.1)	4.8 (4.6, 5.3)	0.001
Glucose 30 min	7.6 (6.7, 8.6)	8 (7.15, 8.9)	<0.001
Glucose 60 min	7 (5.8, 8.4)	7.4 (5.9, 9)	0.042
Glucose 90 min	5.9 (4.9, 7.25)	5.9 (4.8, 7.2)	0.599
Glucose 120 min	5.5 (4.7, 6.5)	5 (4, 6)	<0.001
Glucose 150 min	4.6 (3.7, 5.5)	4 (3.4, 4.75)	<0.001
Glucose 180 min	3.9 (3.4, 4.5)	3.9 (3.6, 4.3)	0.705
C-peptide 0 min [nmol/L]	0.59 (0.48, 0.76)	0.58 (0.45, 0.79)	0.249
C-peptide 30 min	2.05 (1.67, 2.58)	2.18 (1.74, 2.74)	0.015
C-peptide 60 min	2.69 (2.21, 3.36)	2.74 (2.23, 3.51)	0.327
C-peptide 90 min	2.57 (2.11, 3.25)	2.58 (1.91, 3.42)	0.476
C-peptide 120 min	2.23 (1.76, 2.9)	1.89 (1.38, 2.71)	<0.001
C-peptide 150 min	1.64 (1.23, 2.18)	1.16 (0.81, 1.71)	<0.001
C-peptide 180 min	1.06 (0.8, 1.47)	0.75 (0.575, 1.13)	<0.001
Insulin 0 min [mIU/L]	6.1 (4.4, 9.25)	6.2 (4.3, 9.55)	0.443
Insulin 30 min	47.6 (33, 69.8)	45.9 (32.8, 65.1)	0.359
Insulin 60 min	51.5 (35.6, 75)	48.4 (30.2, 74.1)	0.018
Insulin 90 min	39.5 (27.8, 59.9)	35.7 (21.4, 57)	0.001
Insulin 120 min	28.9 (18.4, 44.9)	17.5 (10.2, 32.3)	<0.001
Insulin 150 min	14.3 (7.64, 26.7)	7 (3.9, 15.4)	<0.001
Insulin 180 min	6.4 (4, 11.8)	4.2 (2.76, 7.05)	<0.001
AUC gluc 30 min	185 (170, 203)	192 (179, 212)	<0.001
AUC gluc	1050 (926, 1200)	1040 (914, 1190)	0.733
ΔAUC gluc	207 (110, 341)	184 (88.9, 298)	0.036
AUC ins 30 min	4960 (3420, 7070)	4660 (3470, 6820)	0.367
AUC ins	34,800 (25,900, 50,400)	29,500 (20,000, 47,000)	<0.001
ΔAUC ins	28,000 (20,200, 41,400)	22,400 (15,900, 38,400)	<0.001
AUC cp 30 min	40,200 (32,900, 50,000)	42,900 (34,200, 52,400)	0.055
AUC cp	362 × 10^3^ (307, 447) × 10^3^	338 × 10^3^ (281, 445) × 10^3^	0.011
ΔAUC cp	255 × 10^3^ (207, 318) × 10^3^	235 × 10^3^ (181, 316) × 10^3^	<0.001
**(c)**			
	**Women** **n = 1035**	**Men** **n = 227**	***p*** **-Level**
Insulin Sensitivity/Resistance			
HOMA-R	1.3 (0.89, 2.04)	1.33 (0.9, 2.1)	0.915
QUICKI	0.368 (0.343, 0.391)	0.366 (0.341, 0.39)	0.944
OGIS 2h	458 (418, 497)	457 (410, 500)	0.578
OGIS 3h	505 (453, 550)	486 (440, 527)	<0.001
ISIcomp	8.37 (5.42, 11.2)	8.96 (5.28, 12.5)	0.024
MCRest	9.86 (8.31, 10.9)	9.91 (8.77, 10.7)	0.988
Si(oral)	0.154 (0.0845, 0.254)	0.148 (0.0753, 0.261)	0.925
PREDIM	6.92 (5.35, 8.78)	7.01 (5.46, 8.69)	0.908
Beta Cell Function			
HOMA-beta	104 (73.3, 157)	101 (65.6, 140)	<0.001
Ins0/Gluc0	7.65 (5.73, 11.5)	7.83 (5.44, 11.3)	0.195
Cp0/Gluc0	422 (316, 634)	432 (300, 623)	0.195
IGI	91.6 (58.9, 152)	80.5 (53, 128)	0.005
IGI simplified	38.5 (26.2, 55.2)	34.8 (24.1, 50.4)	0.039
IGI cp	522 (367, 751)	530 (372, 717)	0.935
IGI simplified cp	2120 (1450, 3050)	1920 (1330, 2780)	0.034
AUC ins/AUC gluc	33.4 (25.4, 47)	29 (21.2, 41.8)	<0.001
ΔAUC ins/ΔAUC gluc	159 (93.3, 287)	149 (85, 271)	0.972
AUC cp/AUC gluc	351 (290, 421)	334 (274, 412)	0.014
ΔAUC cp/ΔAUC gluc	1320 (846, 2220)	1380 (885, 2410)	0.214
Disposition Indices			
IGI × ISIcomp	268 (213, 338)	254 (194, 316)	0.016
OGIS 3h × AUCins	17.5 × 10^6^ (13.6, 24.5) × 10^6^	14.4 × 10^6^ (10.1, 21.4) × 10^6^	<0.001
Adaptation Index			
OGIS 3h × AUCcp	18.4 × 10^7^ (15.6, 21.9) × 10^7^	16.3 × 10^7^ (13.9, 20.9) × 10^7^	<0.001
Hepatic Extraction			
HE	67.7 (60.6, 73.2)	70.2 (63.1, 76.8)	<0.001

Data are given as median (95% LCL; 95% UCL) and *p*-levels according to ANOVA test. Explanatory notes and abbreviations for the tabulated parameters are available in Appendix A, Table A1.

## Data Availability

Original data presented in the study are available upon reasonable request to the corresponding author. The data are not publicly available due to privacy policy.

## Appendix A

**Table A1:** Units and explanatory notes to the parameters used in Table 1, Table 2, Table 3, Table 4, Table 5 and Table 6.

Parameter	Details	Units
NGT	normal gulucose tolerance	n/a
IFG + IGT	impaired fasting glucose and/or impaired glucose tolerance	n/a
T2DM	type 2 diabetes mellitus	n/a
IGR	impaired glucose regulation (IFG and/or IGT or T2DM)	n/a
PCOS	women diagnosed with polycystic ovary syndrome	n/a
GDM history	women with positive history of gestational diabetes mellitus	n/a
BMI	body mass index	kg/m^2^
BAI	body adiposity index; BAI = ((hip circumference)/((height)^1.5^) − 18)	%
WHR	waist to hip ratio	dimensionless
TSH	thyroid-stimulating hormone	mIU/L
Free T4	free triiodothyronine	pmol/L
Free T3	free thyroxine	pmol/L
ALT	alanine aminotransferaase	ukat/L
AST	aspartate transferase	ukat/L
GGT	gamma-glutamyltransferase	ukat/L
OGTT Description		
Glucose	Glucose concentration in individual minutes of OGTT	mmol/L
Insulin	Insulin concentration in individual minutes of OGTT	mIU/L
C-peptide	C-peptide concentration in individual minutes of OGTT	nmol/L
AUC gluc 30 min	AUC of glucose in the first 30 min	mmol/L × min
AUC gluc	AUC of glucose (total)	mmol/L × min
ΔAUC gluc	Incremental (suprabasal) AUC of glucose	mmol/L × min
AUC ins 30 min	AUC of insulin in the first 30 min	pmol/L × min
AUC ins	AUC of insulin (total)	pmol/L × min
ΔAUC ins	Incremental (suprabasal) AUC of insulin	pmol/L × min
AUC cp 30 min	AUC of C-peptide in the first 30 min	pmol/L × min
AUC cp	AUC of C-peptide (total)	pmol/L × min
ΔAUC cp	Incremental (suprabasal) AUC of C-peptide	pmol/L × min
Insulin Sensitivity/Resistance		
HOMA-R	HOMA-insulin resistance	dimensionless
QUICKI	QUICKI index of insulin sensitivity	dimensionless
OGIS 2h	OGIS index of insulin sensitivity calculated from 2-h OGTT	mL/min/m^2^
OGIS 3h	OGIS index of insulin sensitivity calculated from 3-h OGTT	mL/min/m^2^
ISIcomp	Index of insulin sensitivity also known as Matsuda’s index	[(mg/dL)^2^∙(μU/mL)^2^] ^−1/2^
MCRest	Index of insulin sensitivity also known as Stumvoll’s index	mL/min/kg
Si(oral)	Index of insulin sensitivity also known as Caumo’s index	(mL/min/kg)/(μU/mL)
PREDIM	PREDIM (predictor of clamp-based insulin sensitivity)	mg/min/kg
Beta Cell Function		
HOMA-beta	HOMA-beta cell function	%
Ins0/Gluc0	Ratio of basal insulin and glucose	pmol/mmol
Cp0/Gluc0	Ratio of basal C-peptide and glucose	pmol/mmol
IGI	Insulinogenic index ((Ins30-Ins0)/(Gluc30-Gluc0))	pmol/mmol
IGI simplified	Ins30min/Gluc30min	pmol/mmol
IGI cp	Insulinogenic index with C-peptide ((Cp30-Cp0)/(Gluc30-Gluc0))	pmol/mmol
IGI simplified cp	Cp30min/Gluc30min	pmol/mmol
AUC ins/AUC gluc	Ratio of AUC ins and AUC gluc	pmol/mmol
ΔAUC ins/ΔAUC gluc	Ratio of ΔAUC ins and ΔAUC gluc	pmol/mmol
AUC cp/AUC gluc	Ratio of AUC cp and AUC gluc	pmol/mmol
ΔAUC cp/ΔAUC gluc	Ratio of ΔAUC cp and ΔAUC gluc	pmol/mmol
Disposition Indices		
IGI × ISIcomp	Oral disposition index (one possible formulation)	(see corresponding units)
OGIS 3h × AUCins	Oral disposition index (one possible formulation)	(see corresponding units)
Adaptation Index		
OGIS 3h × AUCcp	Oral adaptation index (one possible formulation)	(see corresponding units)
He	Hepatic insulin extraction	%
